# DNA methylation, nucleic acid structure, and rett mutations tune MeCP2 binding affinity and cooperativity

**DOI:** 10.1016/j.jbc.2026.113227

**Published:** 2026-06-04

**Authors:** Manana Melikishvili, Matthew Rea, Colt Capan, Hyoungjoo Lee, Darrell P. Chandler, Yvonne N. Fondufe-Mittendorf

**Affiliations:** 1 Department of Epigenetics, Van Andel Research Institute, Grand Rapids, Michigan, USA; 2 Mass Spectrometry Core, Van Andel Research Institute, Grand Rapids, Michigan, USA; 3 Department of Cell Biology, Van Andel Research Institute, Grand Rapids, Michigan, USA

**Keywords:** cooperativity, DNA binding protein, DNA methylation, DNA-protein interaction, gene regulation, genetic polymorphism;, methyl-CpG binding protein 2

## Abstract

Methyl-CpG-binding protein 2 (MeCP2) is a chromatin-associated factor whose dysfunction causes Rett syndrome. Although MeCP2 preferentially binds methylated DNA, its affinity for methylated substrates is only ∼ threefold higher than for unmethylated DNA, raising the question of how MeCP2 selectively recognizes its targets. Here, we quantify binding of full-length WT MeCP2 and Rett-associated variants (R106W, T158M, R270X, and R306C) to nucleic acid substrates that vary in length, secondary structure, methylation pattern, CpG symmetry, and strand composition. WT and mutant MeCP2 preferentially bind double-stranded DNA but also interact with single- and double-stranded DNA and RNA with nanomolar affinity. Binding to single-stranded targets is largely driven by the formation of local duplex structures, whereas 5-methylcytosine provides stabilizing contacts and enhances MeCP2 affinity when canonical duplex geometry is absent. Rett-associated mutations segregate into mechanistic classes: mutations within the methyl-CpG-binding domain (R106W and T158M) weaken methylation-dependent recognition and reduce binding affinity, whereas C-terminal mutations (R270X and R306C) preserve high-affinity binding but diminish cooperative interactions consistent with impaired higher-order bridging. These findings identify MeCP2 as a methyl-sensitive nucleic acid binder whose interactions are shaped by local nucleic acid topology and modulated by cytosine methylation, providing a mechanistic framework for understanding how Rett-associated mutations disrupt chromatin regulation.

Rett syndrome is a rare, X-linked neurodevelopmental disorder characterized by a period of normal development until approximately 18 months of age, after which motor and verbal skills begin to regress (1, 2, 3, 4, 5). Affected children may have varying degrees of intellectual and physical disability, autism-like symptoms, hand-wringing, and shortened life expectancies (4). More than 95% of cases are caused by mutations in methyl-CpG-binding protein 2 (MeCP2) (1, 6, 7), a transcriptional regulator that has both activating and repressive functions (3, 8, 9, 10, 11, 12).

MeCP2 is an intrinsically disordered protein containing six domains (Fig. 1), of which only the methyl-CpG-binding domain (MBD) is intrinsically structured. The most frequent MeCP2 mutations occur within the MBD, intervening domain, and transcriptional-repression domain (TRD). Each of these domains is involved in DNA binding and modulating gene transcription (1, 13), but the MBD and TRD are also involved in methyl group binding and co-repression (14, 15). There is increasing evidence that mutations in the DNA-binding domains correlate with clinical symptoms and disease severity (5, 6, 16), but the discovery of mutations throughout the *MeCP2* gene and observations that other MeCP2 domains bind DNA nonspecifically have called this simple view into question (17, 18). Based on these factors, it is now thought that mutations at the N-terminus correlate with severe disease, while mutations at the C-terminus are associated with milder symptoms (19).

**Figure 1:**
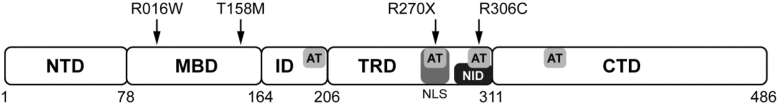
**MeCP2 domains and full-length variants.** N-terminal domain; methyl-CpG-binding domain; intervening domain; = transcriptional repression domain; C-terminal domain. AT nucleotide hook domain. NID = NCoR/SMRT interaction domain. Amino acid numbering (from 1-486) is shown at the *bottom*. Specific amino acid variants used in this study are shown at the *top*. MeCP2, Methyl-CpG-binding protein 2.

MeCP2 was initially thought to bind only methylated cytosine (5 mC) at CpG sites (20, 21, 22, 23), but was later found to bind methylated A and T bases (24, 25) and non-methylated and hydroxymethylated CpG sites (9, 24, 25, 26, 27, 28). Previous biochemical studies indicate that full-length MeCP2 exhibits only a modest, assay-dependent preference for methylated over unmethylated DNA, while the isolated MBD shows substantially greater methylation-dependent selectivity under conditions that reduce nonspecific DNA binding (14, 29, 30). These data raise important questions about how MeCP2 identifies its target sites, and how differential MeCP2 binding at these sites influences transcription (31, 32).

Most MeCP2 binding studies focus on the MBD and MBD mutants, but ignoring the unstructured MeCP2 domains may overlook important allosteric contributions to MeCP2 binding to nucleic acids and chromatin. For example, the MBD binds non-methylated GT-rich sequences *in vitro* (33), and truncated MeCP2 (amino acids 1–205) binds GTGT with high affinity (34), but isolated MBD and full-length MeCP2 bind with reduced affinity (34). Others showed that adding seven amino acids to the C-terminus of the minimal MBD reduced methyl-dependent DNA binding and increased non-specific interactions with DNA (14). However, a recent structure of the low-affinity (∼4 μM) complex between CAC (the complement of GTG) and the minimal MBD could not explain why and how these extra amino acids facilitate binding of the 1 to 205 fragment, or why the full-length protein has a lower binding affinity (33). Still others have suggested that GC-content rather than DNA methylation is the primary determinant of MeCP2 binding (35).

What seems clear from these disparate reports is that MeCP2 binding to DNA is not solely determined by the MBD domain or by DNA methylation, but is also modulated by intrinsically disordered regions. Prior *in vitro* studies differ in target DNA length, methylation state, and protein or domain composition, making it difficult to distinguish high-affinity, context-dependent interactions that are likely to occur under physiological conditions from weaker, non-specific DNA binding observed under experimental conditions. The objective of this study was therefore to measure binding affinities and stoichiometry for WT and mutated full-length MeCP2 proteins with DNA targets of defined size, sequence, and 5 mC content, to identify interaction modes most consistent with MeCP2 function *in vivo*. Such studies are essential for understanding disease pathology and developing small-molecule therapeutics that can discriminate between WT and mutant MeCP2 binding events.

## Results

### MeCP2 binds single-stranded DNA with high affinity in a sequence- and structure-dependent manner

**Figure 2:**
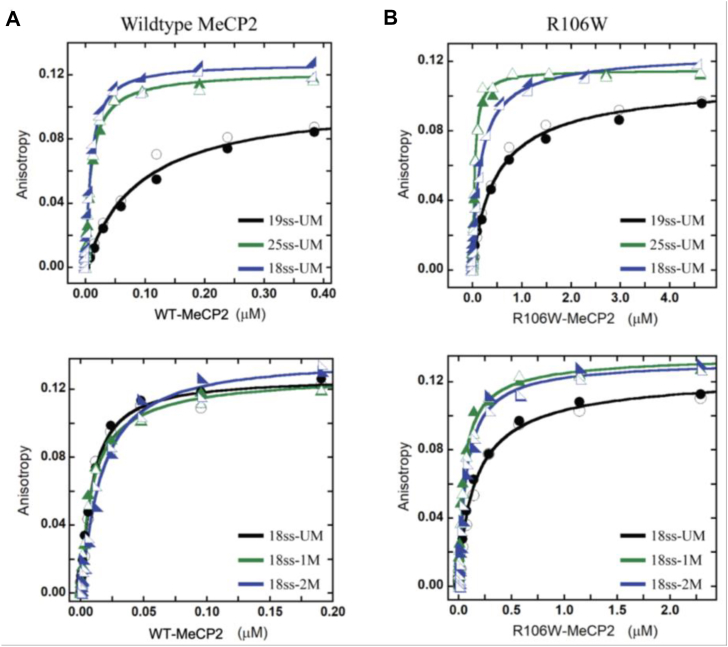
**MeCP2 binding to single-stranded DNA of different lengths and secondary structures.***A,* Binding isotherms for WT MeCP2. Oligonucleotide sequences and dissociation constants are given in Table 1. The -1M and -2M oligonucleotide variants were designed to disrupt the intrinsic stem-loop structure shown in Fig. S10 and demonstrate that WT MeCP2 binds ssDNA in a length- and structure-dependent manner. *B,* same as *A,* except for the R106W MeCP2 variant. The R106W mutant exhibits substantially reduced binding relative to WT MeCP2 but shows similar binding isotherms across oligonucleotide substrates. Data are the mean ± s.d. from three independent experiments. MeCP2, Methyl-CpG-binding protein 2.

The MBD recognizes the minor groove of duplex DNA and relies on base pairing and groove geometry for tight association (36, 37). Because most early *in vitro* studies focused on isolated MBD constructs, MeCP2 has long been considered to interact weakly with single-stranded DNA (38). However, full-length MeCP2 contains additional DNA- and RNA-binding regions outside the MBD, many of which are intrinsically disordered and can mediate nucleic acid interactions independently of CpG methylation (39, 40). To test this, we used fluorescence anisotropy to compare MeCP2 binding to ssDNAs of varying lengths (18–25 nt). WT MeCP2 bound ssDNA with nanomolar affinity, with the *K*_*d*_ ranging from 8.7 ± 0.6 nM for the 18-mer to 90.2 ± 13.3 nM for the 19-mer (Tables 1 and S1; Fig. 2). Extending the 19-mer to a 25-mer by adding three nucleotides to both ends resulted in a *K*_*d*_ = 9.5 ± 1.4 nM, and all MeCP2 variants showed an approximately 10-fold increase in affinity for the 25-mer relative to the 19-mer. The apparent Hill coefficients near unity across all samples (*h* ≈ 1) suggest non-cooperative, monomeric binding. The range of *K*_*d*_ values suggests that DNA length influences binding strength, and that even slightly longer ssDNA substrates enable MeCP2 to establish multiple stabilizing contacts. This is consistent with saturation experiments showing cooperative binding with an apparent stoichiometry of ∼1 MeCP2 molecule per 11 bp of duplex DNA (37), and with the reported MBD footprint (38).

**Table 1:** Binding affinity (*K*_*d*_*)* and cooperativity (h) for MeCP2 variants and single-stranded oligonucleotide targets

Name	ID	Sequence	WT	R270X	R306C	T158M	R106W
18ss-UM	1	FAM–5′–GTTGCCGCGTGGTGGCAG	Kd = 8.7 ± 0.6 h = 1.13 ± 0.08	Kd = 3.7 ± 0.3 h = 1.15 ± 0.08	Kd = 8.2 ± 0.4 h = 1.13 ± 0.05	Kd = 48.8 ± 4.2 h = 0.87 ± 0.05	Kd = 166 ± 17 h = 0.85 ± 0.05
18ss-1M	2	FAM–5′–GTTGCCGCGTGGTG**C**CAG	*K*_*d*_ = 9.7 ± 0.6 h = 0.90 ± 0.04	*K*_*d*_ = 4.7 ± 0.3 h = 1.18 ± 0.08	*K*_*d*_ = 8.2 ± 0.4 h = 0.92 ± 0.04	*K*_*d*_ = 27.1 ± 1.4 h = 0.77 ± 0.02	*K*_*d*_ = 49.2 ± 5.1 h = 0.79 ± 0.05
18ss-2M	3	FAM–5′–GTTGCCGC**C**TGGTG**C**CAG	*K*_*d*_ = 15.9 ± 1.2 h = 1.12 ± 0.07	*K*_*d*_ = 6.9 ± 0.5 h = 1.15 ± 0.08	*K*_*d*_ = 16.5 ± 1.7 h = 1.09 ± 0.10	*K*_*d*_ = 32.9 ± 2.0 h = 0.87 ± 0.04	*K*_*d*_ = 74.0 ± 7.3 h = 0.93 ± 0.06
19ss-UM	4	FAM–5′–AGGCCGCCAGAGAGCGCCC	*K*_*d*_ = 90.2 ± 13.3 h = 1.00 ± 0.09	*K*_*d*_ = 63.0 ± 13.5 H = 0.77 ± 0.07	*K*_*d*_ = 107.7 ± 13.1 h = 1.01 ± 0.08	*K*_*d*_ = 251 ± 39 h = 0.80 ± 0.06	*K*_*d*_ = 497 ± 61 h = 0.89 ± 0.06
25ss-UM	5	FAM–5′–GCTAGGCCGCCAGAGAGCGCCCAAC	*K*_*d*_ = 9.5 ± 1.4 h = 0.91 ± 0.08	*K*_*d*_ = 5.2 ± 0.4 h = 1.00 ± 0.06	*K*_*d*_ = 11.1 ± 2.1 h = 0.83 ± 0.08	*K*_*d*_ = 17.9 ± 2.0 h = 1.04 ± 0.09	*K*_*d*_ = 43.2 ± 4.6 h = 1.03 ± 0.08
25ss-1M	6	FAM–5′–GCTAGGCCGCC**G**GAGAGCGCCCAAC	*K*_*d*_ = 6.5 ± 0.6 h = 1.15 ± 0.09	*K*_*d*_ = 4.7 ± 0.6 h = 0.86 ± 0.06	*K*_*d*_ = 13.1 ± 1.9 h = 1.02 ± 0.10	*K*_*d*_ = 18.9 ± 1.6 h = 0.95 ± 0.06	*K*_*d*_ = 56.6 ± 6.0 h = 0.88 ± 0.05

Binding affinities (*K*_*d*_) are in nM; h = Hill coefficient. Data represent mean ± s.d. from three independent experiments. Base modifications mentioned in the text are in bold. Potential secondary structures are shown in Fig. S10.

UM, unmethylated; ss, single-stranded; ds, double-stranded; 1M, one modification; 2M, two modifications; MeCP2, Methyl-CpG-binding protein 2; FAM, fluorescein amidite.

We observed that the ssDNA 18-mer contained a self-complementary region that could form a stable hairpin, and that the 25-mer could self-anneal (Fig. S10, *A* and *C*). In contrast, the 19-mer lacked complementary motifs and was predicted to remain unstructured. The correlation between predicted secondary structure and measured binding affinity suggests that MeCP2 favors partially folded or structured ssDNA. To test if local secondary structure contributes to MeCP2 binding, we therefore modified the 18-mer ssDNA to disrupt self-complementarity and hairpin structure (Fig. S10*B*). One substitution yielded binding affinities similar to the original target (*K*_*d*_ = 9.7 ± 0.6 nM), whereas two substitutions reduced MeCP2 binding affinity (*K*_*d*_ = 15.9 ± 1.2 nM; Table 1, Fig. 2*A*). Interestingly, R270X and R306C MeCP2 variants behaved like WT MeCP2, while MBD variants T158M and R106W showed enhanced binding to the modified (and unstructured) oligonucleotides (*K*_*d*_ = 27.1–32.9 nM and 49.2–74.0 nM, respectively; Table 1, Fig. 2*B*). These results indicate that secondary structure drives MeCP2 interactions with ssDNA.

The R270X truncated variant exhibited the highest affinity for all 18-mer ssDNA substrates (*K*_*d*_ = 3–7 nM), suggesting that loss of the C-terminal domain relieves an autoinhibitory constraint and enhances DNA association, consistent with prior studies indicating that distal MeCP2 regions modulate nucleic acid binding. On the other hand, the R306C variant exhibited binding affinities similar to those of WT, suggesting that R306C does not contribute to DNA binding. The MBD mutants (T158M and R106W) bound ssDNA with weaker affinities than WT MeCP2 (*K*_*d*_ = 17.9–251 nM and 43–497 nM, respectively; Table 1), indicating that these mutations disrupt the MeCP2-DNA recognition interface. Collectively, these results demonstrate that MeCP2 binds ssDNA with high affinity and that this binding depends on local structural features. Self-complementary motifs that generate duplex structures enhance MeCP2–DNA association, whereas disrupting the local duplex structure reduces affinity. Enhanced binding by R270X indicates that the C-terminal domain negatively regulates ssDNA binding, whereas MBD mutations destabilize DNA recognition.

### MeCP2 prefers double-stranded targets

**Figure 3:**
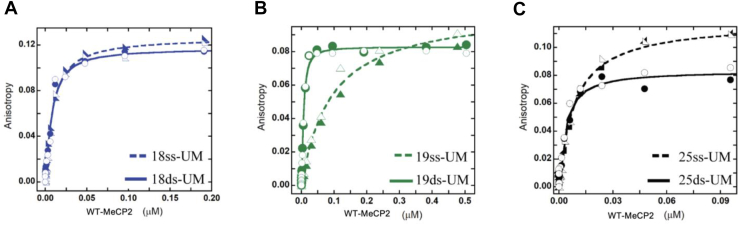
**Local secondary structure influences MeCP2 binding to single- and double-stranded DNA.***A,* binding isotherms for WT MeCP2 and single- or double-stranded 18-mers that can adopt a stable secondary structure. Oligonucleotide sequences and dissociation constants are given in Table 1, and predicted secondary structures are shown in Fig. S10. *B,* same as *A,* except for the unstructured 19-mer oligonucleotides. *C,* Same as *A,* except for 25-mer oligonucleotides. Data are the mean ± s.d. from three independent experiments. A similar trend is observed for MeCP2 mutants (see Table 2). MeCP2, Methyl-CpG-binding protein 2.

MeCP2 is best known for binding double-stranded DNA containing symmetrically methylated CpG dinucleotides (37), but more recent studies show that MeCP2 can also bind methylated CpA, CpC, and CpT dinucleotides that are abundant in neuronal genomes (41, 42). Interestingly, WT MeCP2 bound single- and double-stranded 18-mer DNA with nanomolar affinity, although dsDNA binding was generally stronger (Tables 2 and S2; Fig. 3*A*). However, WT MeCP2 bound the non-structured 19-mer ssDNA with *K*_*d*_ = 90.2 ± 13.3 nM, which improved to 6.3 ± 0.3 nM in the corresponding dsDNA (Table 2, Fig. 3*B*). MeCP2 binding to the 25-mer also increased from 9.5 ± 1.4 nM for ssDNA to 3.7 ± 0.5 nM for dsDNA (Table 2, Fig. 3*C*). Thus, MeCP2 binds single- and double-stranded substrates, with a consistent preference for duplex DNA.

**Table 2:** Binding affinities and Hill coefficients for MeCP2 variants, single-stranded DNA substrates, and their corresponding dsDNAs

Name	ID	Sequence	WT	R270X	R306C	T158M	R106W
18ss-UM	1	FAM–5′–GTTGCCGCGTGGTGGCAG	*K*_*d*_ = 8.7 ± 0.6 h = 1.13 ± 0.08	*K*_*d*_ = 3.7 ± 0.3 h = 1.15 ± 0.08	*K*_*d*_ = 8.2 ± 0.4 h = 1.13 ± 0.05	*K*_*d*_ = 48.8 ± 4.2 h = 0.87 ± 0.05	*K*_*d*_ = 166 ± 17 h = 0.85 ± 0.05
18ds-UM	7	FAM–5′–GTTGCCGCGTGGTGGCAG CAACGGCGCACCACCGTC-5′	*K*_*d*_ = 7.3 ± 0.5 h = 1.75 ± 0.17	*K*_*d*_ = 5.0 ± 0.3 h = 1.46 ± 0.10	*K*_*d*_ = 6.5 ± 0.5 h = 1.73 ± 0.21	*K*_*d*_ = 7.7 ± 0.7 h = 0.80 ± 0.05	*K*_*d*_ = 29.5 ± 2.9 h = 0.77 ± 0.04
19ss-UM	4	FAM–5′–AGGCCGCCAGAGAGCGCCC	*K*_*d*_ = 90.2 ± 13.3 h = 1.00 ± 0.09	*K*_*d*_ = 63.0 ± 13.5 h = 0.77 ± 0.07	*K*_*d*_ = 107.7 ± 13.1 h = 1.01 ± 0.08	*K*_*d*_ = 251 ± 39 h = 0.80 ± 0.06	*K*_*d*_ = 497 ± 61 h = 0.89 ± 0.06
19ds-UM	8	FAM–5′–AGGCCGCCAGAGAGCGCCC TCCGGCGGTCTCTCGCGGG-5′	*K*_*d*_ = 6.3 ± 0.3 h = 1.44 ± 0.09	*K*_*d*_ = 7.5 ± 1.2 h = 0.85 ± 0.09	*K*_*d*_ = 6.6 ± 0.4 h = 1.57 ± 0.12	*K*_*d*_ = 15.1 ± 1.2 h = 0.85 ± 0.04	*K*_*d*_ = 38.9 ± 4.5 h = 0.78 ± 0.05
25ss-UM	5	FAM–5′–GCTAGGCCGCCAGAGAGCGCCCAAC	*K*_*d*_ = 9.5 ± 1.4 h = 0.91 ± 0.08	*K*_*d*_ = 5.2 ± 0.4 h = 1.00 ± 0.06	*K*_*d*_ = 11.1 ± 2.1 h = 0.83 ± 0.08	*K*_*d*_ = 17.9 ± 2.0 h = 1.04 ± 0.09	*K*_*d*_ = 43.2 ± 4.6 h = 1.03 ± 0.08
25ds-UM	10	FAM–5′–GCTAGGCCGCCAGAGAGCGCCCAAC CGATCCGGCGGTCTCTCGCGGGTTG	*K*_*d*_ = 3.7 ± 0.5 h = 1.08 ± 0.12	*K*_*d*_ = 5.0 ± 1.0 h = 0.71 ± 0.06	*K*_*d*_ = 3.2 ± 0.4 h = 1.14 ± 0.13	*K*_*d*_ = 6.2 ± 0.8 h = 1.02 ± 0.10	*K*_*d*_ = 9.4 ± 1.1 h = 0.91 ± 0.07

Binding affinities (*K*_*d*_) are in nM; h = Hill coefficient. Data represent mean ± s.d. from three independent experiments. Some ssDNA data from Table 1 are repeated here to facilitate side-by-side comparisons.

UM, unmethylated; ss, single-stranded; ds, double-stranded; MeCP2, Methyl-CpG-binding protein 2; FAM, fluorescein amidite.

Since the hairpin-prone single-stranded 18-mer and self-annealing 25-mer showed stronger binding than the unstructured 19-mer (Tables 2 and S2), this suggests that MeCP2 does not require a perfect duplex to enhance binding affinity. Rett-associated mutants also preferred dsDNA, but, as before, showed domain-specific differences. The truncated R270X variant bound ssDNA and dsDNA most tightly, indicating that the C-terminal domain negatively regulates dsDNA binding. R306C maintained WT-like affinity, whereas T158M and R106W showed impaired binding and reduced cooperativity relative to WT MeCP2 (Tables 2 and S2). Together, these findings show that MeCP2 binds unmodified dsDNA and partially folded ssDNA with high affinity, recognizing general features of DNA topology. While duplex DNA is the preferred substrate, structured ssDNA can mimic duplex geometry and support stable MeCP2 binding.

### DNA methylation enhances MeCP2 binding to DNA targets, with binding affinity influenced by methyl-group spacing

The MeCP2 methyl-CpG-binding domain (MBD) recognizes 5 mC at CpG dinucleotides in double-stranded DNA (20, 21, 36, 43). However, it remains unclear whether MeCP2 can recognize or bind single-stranded, methylated DNA species that arise during transcription, replication, or DNA repair. Because WT and mutant MeCP2 bind unmodified single-stranded DNA *in vitro* (Tables 1, 2, S1 and S2), we investigated whether the presence, number, and spacing of DNA methylation influence MeCP2 interactions with single-stranded substrates.

**Figure 4:**
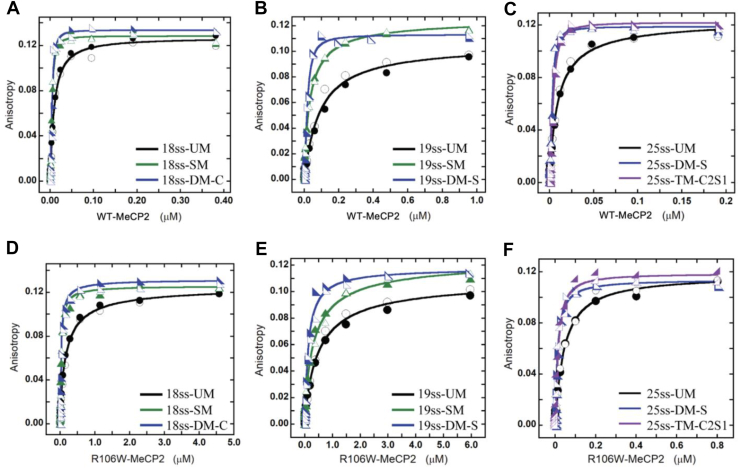
**5 mC number and spacing influence MeCP2 binding affinity to single-stranded DNA.***A,* binding isotherms for WT MeCP2 and single-stranded 18-mers containing 5 mC groups. Oligonucleotide sequences, the position of 5 mC nucleotides, and dissociation constants are given in Table 3. *B*, same as *A,* except for single-stranded 19-mers. *C,* same as *A,* except for single-stranded 25-mers oligonucleotides. *D–F,* same as *A–C,* except with the R106W variant. Data are the mean ± s.d. from three independent experiments. A similar trend is observed for MeCP2 mutants (see Table 3). MeCP2, Methyl-CpG-binding protein 2.

Methyl groups enhanced WT and mutant MeCP2 binding to all ssDNA targets (Tables 3 and S3). For the structured 18-mer, a single methyl group increased WT MeCP2 binding from 8.7 ± 0.6 nM to 3.8 ± 0.2 nM, but adding another methyl group did not further increase WT MeCP2 affinity (Fig. 4*A*). In contrast, one or two methyl groups in the unstructured 19-mer improved MeCP2 binding from 90.2 ± 13.3 nM to 33.6 ± 3.0 nM and 16.6 ± 1.3 nM, respectively (Table 3, Fig. 4*B*). Methyl groups also improved MeCP2 binding to the 25-mer (Fig. 4*C*). Thus, MeCP2 can recognize 5 mC in ssDNA independent of any local structure or duplex DNA.

**Table 3:** Binding affinity (*K*_*d*_*)* and cooperativity (h) for MeCP2 variants and methylated, single-stranded DNA targets

Name	ID	Sequence	WT	R270X	R306C	T158M	R106W
18ss-UM	1	FAM–5′–GTTGCCGCGTGGTGGCAG	*K*_*d*_ = 8.7 ± 0.6 h = 1.13 ± 0.08	*K*_*d*_ = 3.7 ± 0.3 h = 1.15 ± 0.08	*K*_*d*_ = 8.2 ± 0.4 h = 1.13 ± 0.05	*K*_*d*_ = 48.8 ± 4.2 h = 0.87 ± 0.05	*K*_*d*_ = 166 ± 17 h = 0.85 ± 0.05
18ss-SM	11	FAM–5′–GTTGC**C**GCGTGGTGGCAG	*K*_*d*_ = 3.8 ± 0.2 h = 1.60 ± 0.15	*K*_*d*_ = 1.2 ± 0. 1 h = 1.51 ± 0.14	*K*_*d*_ = 3.8 ± 0.3 h = 1.54 ± 0.17	*K*_*d*_ = 6.0 ± 0.4 h = 1.02 ± 0.06	*K*_*d*_ = 25.3 ± 3.5 h = 0.97 ± 0.10
18ss-DM-C	12	FAM–5′–GTTGC**C**G**C**GTGGTGGCAG	*K*_*d*_ = 4.1 ± 0.3 h = 1.80 ± 0.21	*K*_*d*_ = 1.6 ± 0.1 h = 1.50 ± 0.12	*K*_*d*_ = 4.0 ± 0.3 h = 1.60 ± 0.15	*K*_*d*_ = 7.8 ± 0.6 h = 1.08 ± 0.07	*K*_*d*_ = 26.7 ± 3.1 h = 0.90 ± 0.08
19ss-UM	4	FAM–5′–AGGCCGCCAGAGAGCGCCC	*K*_*d*_ = 90.2 ± 13.3 h = 1.00 ± 0.09	*K*_*d*_ = 63.0 ± 13.5 h = 0.77 ± 0.07	*K*_*d*_ = 107.7 ± 13.1 h = 1.01 ± 0.08	*K*_*d*_ = 251 ± 39 h = 0.80 ± 0.06	*K*_*d*_ = 497 ± 61 h = 0.89 ± 0.06
19ss-SM	13	FAM–5′–AGGC**C**GCCAGAGAGCGCCC	*K*_*d*_ = 33.6 ± 3.0 h = 0.88 ± 0.05	*K*_*d*_ = 14.3 ± 1.5 h = 0.96 ± 0.07	*K*_*d*_ = 29.9 ± 2.2 h = 1.02 ± 0.06	*K*_*d*_ = 99.2 ± 9.9 h = 0.92 ± 0.06	*K*_*d*_ = 333 ± 51 h = 0.81 ± 0.06
19ss-DM-S	14	FAM–5′–AGGC**C**GCCAGAGAG**C**GCCC	*K*_*d*_ = 16.6 ± 1.3 h = 1.46 ± 0.13	*K*_*d*_ = 6.8 ± 0.4 h = 1.48 ± 0.11	*K*_*d*_ = 18.7 ± 1.6 h = 1.44 ± 0.14	*K*_*d*_ = 28.0 ± 1.5 h = 1.12 ± 0.06	*K*_*d*_ = 123.7 ± 12.3 h = 1.00 ± 0.07
25ss-UM	5	FAM–5′–GCTAGGCCGCCAGAGAGCGCCCAAC	*K*_*d*_ = 9.5 ± 1.4 h = 0.91 ± 0.08	*K*_*d*_ = 5.2 ± 0.4 h = 1.00 ± 0.06	*K*_*d*_ = 11.1 ± 2.1 h = 0.83 ± 0.08	*K*_*d*_ = 17.9 ± 2.0 h = 1.04 ± 0.09	*K*_*d*_ = 43.2 ± 4.6 h = 1.03 ± 0.08
25ss-1M	6	FAM–5′–GCTAGGCCGCC**G**GAGAGCGCCCAAC	*K*_*d*_ = 6.5 ± 0.6 h = 1.15 ± 0.09	*K*_*d*_ = 4.7 ± 0.6 h = 0.86 ± 0.06	*K*_*d*_ = 13.1 ± 1.9 h = 1.02 ± 0.10	*K*_*d*_ = 18.9 ± 1.6 h = 0.95 ± 0.06	*K*_*d*_ = 56.6 ± 6.0 h = 0.88 ± 0.05
25ss-DM-S	15	FAM–5′–GCTAGGC**C**GCCAGAGAG**C**GCCCAAC	*K*_*d*_ = 2.6 ± 0.2 h = 1.66 ± 0.18	*K*_*d*_ = 1.2 ± 0.1 h = 1.31 ± 0.11	*K*_*d*_ = 2.4 ± 0.3 h = 1.49 ± 0.20	*K*_*d*_ = 2.8 ± 0.3 h = 0.98 ± 0.07	*K*_*d*_ = 11.3 ± 0.9 h = 1.03 ± 0.07
25ss-TM-C2S1	16	FAM–5′–GCTAGGC**C**GC**C**GGAGAG**C**GCCCAAC	*K*_*d*_ = 3.5 ± 0.2 h = 1.64 ± 0.12	*K*_*d*_ = 1.9 ± 0.1 h = 1.49 ± 0.11	*K*_*d*_ = 1.9 ± 0.3 h = 1.59 ± 0.34	*K*_*d*_ = 4.3 ± 0.2 h = 1.36 ± 0.07	*K*_*d*_ = 14.4 ± 1.2 h = 1.19 ± 0.09

Binding affinities (*K*_*d*_) are in nM; h = Hill coefficient. Data represent mean ± s.d. from three independent experiments. Methylated bases (5 mC) within the canonical CpG dyad are underlined. Some ssDNA data from Table 1 are repeated here to facilitate side-by-side comparisons.

UM, unmethylated; ss, single-stranded; ds, double-stranded; SM, singly methylated; DM, doubly methylated; TM, trimethylated; DM-C, doubly methylated (contiguous); DM-S, doubly methylated (spaced); TM-C2S1, trimethylated (two contiguous + one spaced); MeCP2, Methyl-CpG-binding protein 2.

5 mC spacing clearly influenced MeCP2 affinity for ssDNA, especially ssDNA targets lacking secondary structure (Tables 3 and S3). For example, contiguous 5 mC groups in the 18-mer did not improve WT MeCP2 binding affinity (3.8 ± 0.2 nM *versus*. 4.1 ± 0.3 nM; Table 3), suggesting that clustering or hairpin formation compensates for reduced flexibility or steric hindrance. In contrast, spaced 5 mC nucleotides in the non-structured 19-mer enhanced WT MeCP2 binding from 33.6 ± 3.0 nM to 16.6 ± 1.3 nM (sequence 13 *versus*. 14; Table 3). The apparent Hill coefficients for the 18-, 19-, and 25-mer methylated ssDNAs (*h* = 1.2–1.8) were higher than for unmethylated DNA (*h* ≈ 1.0), suggesting positive cooperativity, multivalent 5 mC engagement, or transient MeCP2 domain interactions on extended templates. Most MeCP2 mutants with an intact MBD displayed similar trends, whereas MBD mutations (T158 and R106W) caused binding defects (Tables 3 and S3; Fig. 4, *D*–*F*). Collectively, these data suggest that 5 mC spacing stabilizes the MBD–DNA interface by reducing steric interference between adjacent methyl groups and the MBD binding surface.

**Figure 5:**
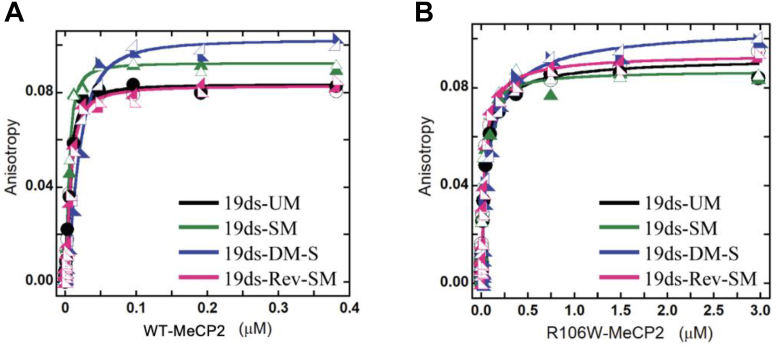
**MeCP2 interactions with dsDNA are governed by DNA length, 5 mC patterning, and MeCP2 domain integrity.***A,* binding isotherms for WT MeCP2 and double-stranded 19-mers containing 5 mC groups. Oligonucleotide sequences, the position of 5 mC nucleotides, and dissociation constants are given in Table 4. *B,* same as *A,* except with the R106W variant. Data are the mean ± s.d. from three independent experiments. Data for all other double-stranded targets and MeCP2 variants are given in Table 4. MeCP2, Methyl-CpG-binding protein 2.

5 mC generally improved WT MeCP2 binding to the 18-, 19-, and 25 bp dsDNAs relative to their non-methylated counterparts (Tables 4 and S4; Fig. 5). Relative to the methylated ssDNA targets (Table 3 and S3), WT MeCP2 binding to the corresponding methylated dsDNA targets was uniformly high and showed little dependence on the presence or spacing of 5 mC groups (Table 4 and S4). However, we were surprised to find that WT binding affinity seemed to depend upon which strand contained the methyl group (*e.g.*, compare WT MeCP2 binding to sequences 17 *versus* 18 and 21 *versus* 22 in Table 4 and S4). There was also no obvious preference for symmetrically methylated DNA (sequence 19 *versus* 17 in Table 4 and S4), in apparent contrast to prior reports demonstrating preferential binding of MeCP2 to symmetrically methylated CpG-containing duplex DNA (37, 38). This discrepancy likely reflects differences in substrate structure and experimental context: earlier studies primarily examined short duplex DNA targets and isolated MBD domains, whereas our measurements were performed with full-length MeCP2 and short single-stranded DNA substrates. Increasing the number of methylated sites did not necessarily increase WT MeCP2 binding affinity relative to a single methyl group (*e.g.*, sequence 23 *versu*s 21 in Table 4 and S4), even though structural models show that the MeCP2 MBD can accommodate two methyl groups (36). Consistent with the data in Table 2, WT MeCP2 displayed higher affinity for the longer 25 bp, methylated dsDNA substrates relative to the 18- and 19-mers (compare sequences 10, 24–26 to 7, 17–20 in Table 4 and S4), despite equivalent CpG content in the 19- and 25-mers. The R106W variant exhibited reduced binding affinity relative to WT MeCP2 and the other variants, consistent with impaired primary DNA recognition. In contrast, all Rett-associated mutants showed reduced cooperativity relative to WT MeCP2 (*e.g.*, Fig. 5*B*), indicating defects in higher-order assembly on dsDNA substrates. However, the R306C variant sometimes had increased cooperativity relative to WT MeCP2 under select conditions (Table 4 and S4). Together, these results indicate that MeCP2 interactions with dsDNA are governed by DNA length, 5 mC patterning, and MeCP2 domain integrity, which together determine binding affinity and the mode of cooperative assembly.

**Table 4:** Binding affinity (*K*_*d*_*)* and cooperativity (h) for MeCP2 variants and double-stranded oligonucleotide targets

Name	ID	Sequence	WT	R270X	R306C	T158M	R106W
18ds-UM	7	FAM–5′–GTTGCCGCGTGGTGGCAG CAACGGCGCACCACCGTC-5′	*K*_*d*_ = 7.3 ± 0.5 h = 1.75 ± 0.17	*K*_*d*_ = 5.0 ± 0.3 h = 1.46 ± 0.10	*K*_*d*_ = 6.5 ± 0.5 h = 1.73 ± 0.21	*K*_*d*_ = 7.7 ± 0.7 h = 0.80 ± 0.05	*K*_*d*_ = 29.5 ± 2.9 h = 0.77 ± 0.04
18ds-SM	17	FAM–5′–GTTGC**C**GCGTGGTGGCAG CAACGGCGCACCACCGTC-5′	*K*_*d*_ = 4.9 ± 0.4 h = 1.77 ± 0.21	*K*_*d*_ = 2.5 ± 0.2 h = 1.52 ± 0.15	*K*_*d*_ = 4.2 ± 0.3 h = 2.78 ± 0.41	*K*_*d*_ = 4.1 ± 0.2 h = 1.07 ± 0.05	*K*_*d*_ = 19.0 ± 1.3 h = 0.97 ± 0.05
18ds-Rev-SM	18	FAM–5′–GTTGCCGCGTGGTGGCAG CAACGG**C**GCACCACCGTC-5′	*K*_*d*_ = 7.3 ± 0.3 h = 1.78 ± 0.12	*K*_*d*_ = 5.3 ± 0.2 h = 1.74 ± 0.12	*K*_*d*_ = 7.0 ± 0.5 h = 1.23 ± 0.09	*K*_*d*_ = 7.2 ± 0.6 h = 0.96 ± 0.06	*K*_*d*_ = 27.1 ± 2.7 h = 0.87 ± 0.06
18ds-FM	19	FAM–5′–GTTGC**C**GCGTGGTGGCAG CAACGG**C**GCACCACCGTC-5′	*K*_*d*_ = 4.8 ± 0.4 h = 1.94 ± 0.24	*K*_*d*_ = 2.7 ± 0.2 h = 1.41 ± 0.13	*K*_*d*_ = 5.6 ± 0.1 h = 1.25 ± 0.17	*K*_*d*_ = 3.0 ± 0.2 h = 1.25 ± 0.08	*K*_*d*_ = 8.9 ± 0.7 h = 0.85 ± 0.05
18ds-DM-C	20	FAM–5′–GTTGC**C**G**C**GTGGTGGCAG CAACGGCGCACCACCGTC-5′	*K*_*d*_ = 3.6 ± 0.3 h = 1.73 ± 0.20	*K*_*d*_ = 2.6 ± 0.2 h = 1.30 ± 0.12	*K*_*d*_ = 3.5 ± 0.3 h = 1.76 ± 0.18	*K*_*d*_ = 4.5 ± 0.3 h = 0.91 ± 0.05	*K*_*d*_ = 22.2 ± 3.1 h = 0.79 ± 0.07
19ds-UM	8	FAM–5′–AGGCCGCCAGAGAGCGCCC TCCGGCGGTCTCTCGCGGG-5′	*K*_*d*_ = 6.3 ± 0.3 h = 1.44 ± 0.09	*K*_*d*_ = 7.5 ± 1.2 h = 0.85 ± 0.09	*K*_*d*_ = 6.6 ± 0.4 h = 1.57 ± 0.12	*K*_*d*_ = 15.1 ± 1.2 h = 0.85 ± 0.04	*K*_*d*_ = 38.9 ± 4.5 h = 0.78 ± 0.05
19ds-SM	21	FAM–5′–AGGC**C**GCCAGAGAGCGCCC TCCGGCGGTCTCTCGCGGG-5′	*K*_*d*_ = 4.9 ± 0.3 h = 1.53 ± 0.13	*K*_*d*_ = 4.0 ± 0.4 h = 1.08 ± 0.10	*K*_*d*_ = 5.5 ± 0.4 h = 1.46 ± 0.14	*K*_*d*_ = 7.6 ± 0.4 h = 1.01 ± 0.04	*K*_*d*_ = 23.4 ± 2.4 h = 0.91 ± 0.07
19ds-Rev-SM	22	FAM–5′–AGGCCGCCAGAGAGCGCCC TCCGG**C**GGTCTCTCGCGGG-5′	*K*_*d*_ = 7.0 ± 0.5 h = 1.41 ± 0.12	*K*_*d*_ = 9.0 ± 0.9 h = 0.86 ± 0.05	*K*_*d*_ = 6.4 ± 0.4 h = 1.74 ± 0.15	*K*_*d*_ = 14.0 ± 1.5 h = 0.74 ± 0.04	*K*_*d*_ = 31.2 ± 2.8 h = 0.83 ± 0.05
19ds-DM-S	23	FAM–5′–AGGC**C**GCCAGAGAG**C**GCCC TCCGGCGGTCTCTCGCGGG-5′	*K*_*d*_ = 17.9 ± 0.8 h = 1.67 ± 0.11	*K*_*d*_ = 9.6 ± 0.6 h = 1.29 ± 0.08	*K*_*d*_ = 15.8 ± 0.8 h = 1.72 ± 0.13	*K*_*d*_ = 15.9 ± 0.9 h = 1.04 ± 0.05	*K*_*d*_ = 80.6 ± 8.1 h = 0.78 ± 0.04
25ds-UM	10	FAM–5′–GCTAGGCCGCCAGAGAGCGCCCAAC CGATCCGGCGGTCTCTCGCGGGTTG	*K*_*d*_ = 3.7 ± 0.5 h = 1.08 ± 0.12	*K*_*d*_ = 5.0 ± 1.0 h = 0.71 ± 0.06	*K*_*d*_ = 3.2 ± 0.4 h = 1.14 ± 0.13	*K*_*d*_ = 6.2 ± 0.8 h = 1.02 ± 0.10	*K*_*d*_ = 9.4 ± 1.1 h = 0.91 ± 0.07
25ds-1M	24	FAM–5′–GCTAGGCCGCCGGAGAGCGCCCAAC CGATCCGGCGGCCTCTCGCGGGTTG	*K*_*d*_ = 2.6 ± 0.3 h = 1.40 ± 0.17	*K*_*d*_ = 4.2 ± 1.1 h = 0.72 ± 0.09	*K*_*d*_ = 2.9 ± 0.3 h = 1.32 ± 0.14	*K*_*d*_ = 4.4 ± 0.6 h = 0.78 ± 0.06	*K*_*d*_ = 9.1 ± 1.1 h = 1.05 ± 0.11
25ds-DM-S	25	FAM–5′–GCTAGGC**C**GCCAGAGAG**C**GCCCAAC CGATCCGGCGGTCTCTCGCGGGTTG	*K*_*d*_ = 2.3 ± 0.5 h = 0.90 ± 0.14	*K*_*d*_ = 1.8 ± 0.3 h = 0.68 ± 0.06	*K*_*d*_ = 2.1 ± 0.2 h = 2.10 ± 0.32	*K*_*d*_ = 2.8 ± 0.3 h = 1.01 ± 0.09	*K*_*d*_ = 5.4 ± 0.8 h = 0.85 ± 0.09
25ds-TM-C2S1	26	FAM–5′–GCTAGGC**C**GC**C**GGAGAG**C**GCCCAAC CGATCCGGCGGTCTCTCGCGGGTTG	*K*_*d*_ = 2.4 ± 0.2 h = 1.52 ± 0.14	*K*_*d*_ = 2.7 ± 0.4 h = 0.92 ± 0.10	*K*_*d*_ = 1.7 ± 0.3 h = 1.27 ± 0.23	*K*_*d*_ = 2.6 ± 0.2 h = 1.61 ± 0.18	*K*_*d*_ = 6.9 ± 0.8 h = 0.97 ± 0.09

Binding affinities (*K*_*d*_) are in nM; h = Hill coefficient. Data represent mean ± s.d. from three independent experiments. Methylated bases (5 mC) within the canonical CpG dyad are underlined. Some dsDNA data from Table 2 are repeated here to facilitate side-by-side comparisons.

MeCP2, Methyl-CpG-binding protein 2.

### MeCP2 preferentially binds DNA over RNA

**Figure 6:**
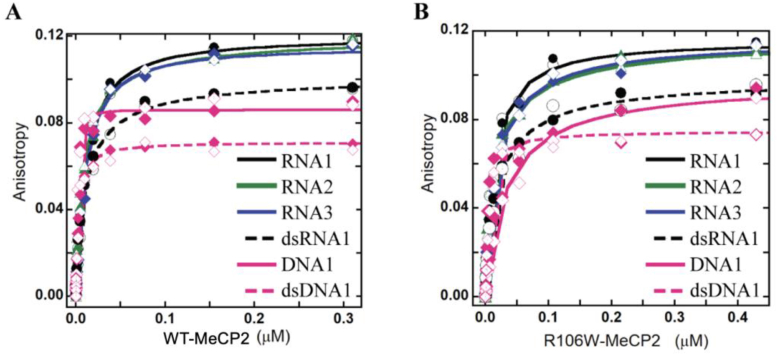
**MeCP2 preferentially binds double-stranded DNA over RNA.***A,* binding isotherms for WT MeCP2 and 26-mer DNA and RNA oligonucleotides. Oligonucleotide sequences and dissociation constants are given in Table 5, and potential secondary structures are given in Fig. S10. *B,* same as *A,* except with the R106W variant. Data are the mean ± s.d. from three independent experiments. MeCP2, Methyl-CpG-binding protein 2.

Prior studies show that MeCP2 can bind RNA (44, 45), with a preference for GC-rich, single-stranded RNAs that adopt extended stem-loop structures (44). We therefore used fluorescence polarization to determine if MeCP2 prefers DNA or RNA substrates with varying secondary structures (Fig. S10, *D*–*F*) and helical grooves (*i.e.*, dsRNA adopts an A-form helix distinct from the B-form dsDNA helix and transiently occurs during transcription and RNA processing). All MeCP2 variants bound the ssRNAs with nanomolar affinities, with a higher affinity for structured RNA1 and RNA2 than for the unstructured RNA3 (compare sequences 27 and 31 to sequence 32 in Tables 5 and S5; Fig. 6). While WT, R270X, and R306C MeCP2 bound DNA1 with higher affinity than the corresponding RNA, the T158M and R106W variants showed reduced discrimination (sequence 27 vs. 28 in Tables 5 and S5), implying that the MeCP2 MBD recognizes RNA. As in Tables 2 and S2, MeCP2 variants bound the dsDNA more tightly than the corresponding ssDNA or ssRNA targets and bound structured RNA with higher affinity than unstructured RNA (Tables 5 and S5). However, binding affinity to the dsRNA1 target was lower than for the corresponding single-stranded RNA1 containing only a short duplex (compare sequence 29–27 in Tables 5 and S5), and lower than for the corresponding dsDNA. The apparent Hill coefficients for all MeCP2 variants and targets (*h* ≈ 1) suggest minimal binding cooperativity. MeCP2 binding to RNA is enhanced by short, structured duplexes, as is MeCP2 binding to ssDNA targets that adopt intrinsic secondary structure. MBD mutations weakened MeCP2 binding to ssRNA, but not to the same extent as for 25- and 26-mer ssDNA targets (see sequence 5 in Tables 2 and S2, and sequence 28 in Tables 5 and S5). Together, these data indicate that MeCP2 variants prefer DNA over RNA and a B-helix over an A-helix, but that all Rett-associated MeCP2 variants bind single- and double-stranded RNA with nanomolar affinity, especially when RNA forms a local duplex structure.

**Table 5:** MeCP2 variant binding affinity for single- and double-stranded RNA and DNA targets

Name	ID	Sequence	WT	R270X	R306C	T158M	R106W
RNA1	27	FAM-5′-GCGGUGUAUAGCCUAAUCUUUACCGC	*K*_*d*_ = 8.6 ± 0.5 h = 1.24 ± 0.08	*K*_*d*_ = 7.6 ± 0.6 h = 0.99 ± 0.06	*K*_*d*_ = 8.3 ± 0.7 h = 1.11 ± 0.08	*K*_*d*_ = 10.3 ± 1.0 h = 0.95 ± 0.07	*K*_*d*_ = 13.0 ± 0.8 h = 0.96 ± 0.05
DNA1	28	FAM-5′-GCGGTGTATAGCCTAATCTTTACCGC	*K*_*d*_ = 2.7 ± 0.2 h = 1.28 ± 0.09	*K*_*d*_ = 2.4 ± 0.1 h = 1.23 ± 0.07	*K*_*d*_ = 2.8 ± 0.2 h = 1.25 ± 0.07	*K*_*d*_ = 11.5 ± 0.7 h = 0.98 ± 0.04	*K*_*d*_ = 34.0 ± 3.9 h = 0.92 ± 0.06
dsRNA1	29	FAM-5′-GCGGUGUAUAGCCUAAUCUUUACCGC CGCCACAUAUCGGAUUAGAAAUGGCG-5′	*K*_*d*_ = 15.8 ± 2.1 h = 0.80 ± 0.06	*K*_*d*_ = 7.9 ± 0.7 h = 1.13 ± 0.10	*K*_*d*_ = 11.3 ± 0.9 h = 0.89 ± 0.05	*K*_*d*_ = 10.5 ± 1.7 h = 0.67 ± 0.05	*K*_*d*_ = 17.7 ± 1.3 h = 0.93 ± 0.05
dsDNA1	30	FAM-5′-GCGGTGTATAGCCTAATCTTTACCGC CGCCACATATCGGATTAGAAATGGCG-5′	*K*_*d*_ = 2.3 ± 0.2 h = 1.18 ± 0.10	*K*_*d*_ = 4.0 ± 0.6 h = 1.00 ± 0.12	*K*_*d*_ = 2.3 ± 0.3 h = 1.17 ± 0.13	*K*_*d*_ = 2.1 ± 0.2 h = 1.02 ± 0.09	*K*_*d*_ = 4.4 ± 0.4 h = 0.99 ± 0.07
RNA2	31	FAM-5′-GCGGUGCGGUGCCUAAUGGGGACCGC	*K*_*d*_ = 9.0 ± 0.7 h = 0.83 ± 0.04	*K*_*d*_ = 6.3 ± 0.5 h = 0.80 ± 0.04	*K*_*d*_ = 9.6 ± 0.7 h = 0.78 ± 0.04	*K*_*d*_ = 12.3 ± 1.2 h = 0.74 ± 0.04	*K*_*d*_ = 15.8 ± 1.0 h = 0.74 ± 0.03
RNA3	32	FAM-5′-CGCAAAAACACAUAAAUCAAAACCAA	*K*_*d*_ = 12.9 ± 1.0 h = 0.95 ± 0.06	*K*_*d*_ = 8.2 ± 0.6 h = 1.19 ± 0.09	*K*_*d*_ = 11.0 ± 0.9 h = 0.90 ± 0.05	*K*_*d*_ = 15.9 ± 1.0 h = 0.82 ± 0.03	*K*_*d*_ = 25.9 ± 1.9 h = 0.94 ± 0.05

Binding affinities (*K*_*d*_) are in nM; h = Hill coefficient. Data represent mean ± s.d. from three independent experiments. Potential secondary structures are shown in Fig. S10.

MeCP2, Methyl-CpG-binding protein 2.

### MeCP2 binding to long DNA substrates

**Figure 7:**
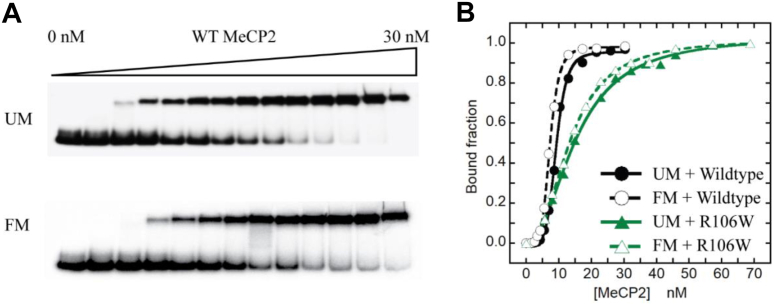
**Cooperative binding of MeCP2 variants to non-methylated and fully methylated 195-bp DNA.***A,* representative electrophoretic mobility shift assays f WT MeCP2 binding to an unmethylated or fully methylated 195 bp DNA fragment. Across all conditions, a single sharp, high-molecular-weight MeCP2–DNA complex is observed. *B,* binding isotherms for WT MeCP2 and the R106W variant. Dissociation constants are given in Table 6. Data are the mean ± s.d. from three independent experiments. MeCP2, Methyl-CpG-binding protein 2.

*In vivo,* MeCP2 acts on chromatin and genomic DNA rather than on oligonucleotides. We therefore tested how MeCP2 variants bind to an unmethylated or fully methylated 195 bp DNA fragment (see Fig. S9). For all MeCP2 variants and both substrates, increasing protein concentrations produced a single, sharp, high–molecular weight MeCP2–DNA complex in electrophoretic mobility shift assay experiments (Figs. 7*A* and S11) and nanomolar dissociation constants (Tables 6 and S6). All MeCP2 variants had improved affinity for the methylated 195 bp target, and *h* > 2 for all variants suggest cooperative MeCP2 binding (Table 6). WT MeCP2 bound approximately 5 to 6 molecules per DNA target, whereas Rett-associated MeCP2 variants bound only 2 to 3 molecules per target (Table 6). This reduced stoichiometry indicates that disease-associated mutations impair multivalent or cooperative MeCP2 assembly on nucleic acids even when DNA binding is retained, and suggests that mutant MeCP2 may not form higher-order nucleoprotein assemblies to modulate chromatin architecture *in vivo*.

**Table 6:** MeCP2 variant binding affinity for a 195 bp, double-stranded DNA

Name	ID	195 bp target	WT	R270X	R306C	T158M	R106W
195ds-UM	33	Unmethylated	*K*_*d*_ = 9.2 ± 0.1 h = 5.79 ± 0.38 *K*_*mono*_ = 1.23 ± 0.08 n = 4.61 ± 0.74	*K*_*d*_ = 5.7 ± 0.2 h = 2.66 ± 0.19 *K*_*mono*_ = 2.84 ± 0.09 n = 2.03 ± 0.12	*K*_*d*_ = 9.0 ± 0.2 h = 2.60 ± 0.12 *K*_*mono*_ = 1.46 ± 0.03 n = 2.87 ± 0.15	*K*_*d*_ = 9.1 ± 0.3 h = 2.11 ± 0.16 *K*_*mono*_ = 1.32 ± 0.05 n = 2.00 ± 0.16	*K*_*d*_ = 15.3 ± 0.4 h = 2.14 ± 0.11 *K*_*mono*_ = 0.82 ± 0.01 n = 2.28 ± 0.04
195ds-FM	34	Fully methylated	*K*_*d*_ = 7.3 ± 0.1 h = 5.31 ± 0.26 *K*_*mono*_ = 1.70 ± 0.09 n = 5.35 ± 0.69	*K*_*d*_ = 5.6 ± 0.2 h = 2.91 ± 0.25 *K*_*mono*_ = 2.82 ± 0.15 n = 2.79 ± 0.50	*K*_*d*_ = 7.7 ± 0.3 h = 2.47 ± 0.19> *K*_*mono*_ = 1.77 ± 0.04 n = 2.89 ± 0.23	*K*_*d*_ = 7.7 ± 0.6 h = 2.28 ± 0.36 *K*_*mono*_ = 1.57 ± 0.12 n = 2.11 ± 0.38	*K*_*d*_ = 13.5 ± 0.3 h = 2.35 ± 0.11 *K*_*mono*_ = 0.92 ± 0.01 n = 2.52 ± 0.05

The DNA sequence and 5 mC modifications are shown in Fig. S9. *K*_*d*_ values are in nM; h = Hill coefficient; *K*_*mono*_ values are in 10^8^ (M^−1^); n = binding stoichiometry. Data are the mean ± s.d. from three independent electrophoretic mobility shift assay experiments.

MeCP2, Methyl-CpG-binding protein 2.

## Discussion

### Structural determinants governing MeCP2 binding affinity

Collectively, this study establishes that MeCP2 and its Rett-associated variants recognize single- and double-stranded DNA and RNA. The data also provide a quantitative and mechanistic framework for how MeCP2 integrates nucleic acid topology, 5 mC patterning, and MeCP2 domain integrity to regulate chromatin architecture, transcription, and genome integrity.

Prior structural studies indicate that the MeCP2 MBD has a defined dsDNA-binding surface, a hydrophobic pocket, and bound water (36, 37, 38). Consistent with this architecture, we find that WT and mutant MeCP2 preferentially bind double-stranded DNA over single-stranded substrates, and that local duplex structure is a dominant determinant of affinity even when the duplex is short or transient (*e.g.*, Tables 1, 2, 5, S1, S2, and S5; secondary structures in Fig. S10). The relative importance of 5 mC to MeCP2 binding affinity depends on nucleic acid context and methylation patterning. In single-stranded DNA, 5 mC enhances MeCP2 binding, particularly for variants with reduced baseline affinity or when methylated cytosines are spaced rather than contiguous (Tables 3 and S3). In contrast, 5 mC presence, spacing, or symmetry did not appreciably increase MeCP2 binding affinity for dsDNA (Tables 4 and S4), especially once the DNA targets reached (or exceeded) 25 bp (Table 4, Table 5, Table 6). Thus, MeCP2 has high intrinsic affinity for backbone/groove complementarity (including ssDNA and RNA with intrinsic secondary structure), and 5 mC-dependent contacts become increasingly important when duplex structures are absent (*e.g.*, unstructured ssDNA). When obvious base-pairing or groove architecture are absent, 5 mC may instead promote favorable local conformations (stacking and transient structure), and/or strengthen sub-optimal DNA contacts *via* hydrogen bonds, electrostatic interactions, or structured water molecules (36, 37).

As DNA substrates increase in length, the relative importance of local duplex structure and methylation diminishes, and cooperative, multivalent interactions dominate. The MeCP2 variant data indicate that stabilizing contacts on extended nucleic acid targets arise beyond the CpG core and involve unstructured MeCP2 domains, particularly the C-terminus. Consistent with prior work, these regions contribute more to multimerization, bridging, and chromatin compaction than to primary site recognition (14, 46). This model aligns with evidence that MeCP2 competes with linker histone H1 at chromatin binding sites (47, 48, 49); engages chromatin features that reinforce stable chromatin association (14, 28, 46, 48); and that Rett-associated MeCP2 mutations disrupt coupling to the NCoR/SMRT corepressor complexes, thereby separating DNA recognition from downstream effector recruitment (10). *In vivo,* MeCP2 distribution tracks global methylation density rather than discrete CpG sites or condensate formation, further supporting a model of topology- and density-dependent chromatin engagement (50, 51). Thus, MeCP2 functions as a chromatin reader that engages nucleic acids through topology-dependent interactions which are influenced by nucleic acid structure, length, CpG density, and chromatin context (47, 49, 52), and *via* MBD methylation-dependent recognition (37).

### Rett syndrome and RNA transcription

MeCP2 links DNA methylation to chromatin accessibility, nucleosome organization, higher-order chromatin structure, and transcriptional output (36, 37), and is central to Rett syndrome pathogenesis and other neurological phenotypes (5, 14, 20, 21, 50, 53, 54, 55, 56, 57, 58). Here, we show that Rett-associated MeCP2 variants segregate into mechanistically informative classes. In general, MBD mutations (R106W, T158M) reduce MeCP2 binding to unmethylated and methylated single- and double-stranded DNA targets, likely by disrupting the primary DNA-recognition surface (36, 37). MBD mutants also exhibit reduced cooperativity for ssDNA, dsDNA, and RNA targets (most evident for the 195 bp target; Table 6), consistent with an impaired ability to nucleate stable complexes on DNA (51). In contrast, the R270X variant consistently increased binding affinity and decreased cooperativity on dsDNA targets relative to WT MeCP2, suggesting that the MeCP2 C-terminal region normally constrains DNA binding and cooperative assembly, or that it exposes additional DNA-interacting surfaces. The R306C variant behaves much like WT MeCP2 on short DNA substrates (Tables 4 and S4) but exhibits reduced cooperativity on extended targets (Table 6).

During transcription, the elongation complex generates transient ssDNA, RNA–DNA hybrids, and R-loops, particularly at long, highly transcribed genes that are disproportionately affected in Rett syndrome (41, 59). These nucleic acid intermediates can alter chromatin structure, impede transcriptional elongation, and promote DNA damage, particularly in post-mitotic neurons (60, 61, 62). Thus, MeCP2 is implicated in transcriptional regulation, RNA processing (40, 63), and R-loop homeostasis (64), processes that require coordinated management of DNA–RNA intermediates. Our findings that MeCP2 binds ssDNA and ssRNA with nanomolar affinity, and especially to substrates with local duplex character, suggest that MeCP2 can engage and stabilize these intermediates *in vivo*. Our finding that 5 mC “compensates” for loss of duplex geometry also provides a putative mechanism by which MeCP2 may remain associated with actively transcribed, methylated DNA regions despite ongoing unwinding and topological stress. Within this framework, we can now rationalize mutation-dependent phenotypes in Rett syndrome. MBD mutations (R106W, T158M) disrupt both methylation-dependent recognition and higher-order assembly, likely destabilizing chromatin at methylated, transcriptionally active loci. In contrast, C-terminal mutations may preserve primary binding while impairing cooperative bridging and multivalent chromatin engagement. More broadly, the ability of MeCP2 to couple DNA methylation state to nucleic acid topology positions it as a key architectural regulator linking epigenetic modification to chromatin folding and genome integrity, and disrupting this coordinated recognition likely underlies the chromatin and transcriptional abnormalities characteristic of Rett syndrome (47, 49).

## Experimental procedures

### Recombinant MeCP2 purification

We selectedWT MeCP2 and four Rett-associated variants, including two with mutations in the methyl-CpG-binding domain (MBD; R106W and T158M), one C-terminal truncation (R270X), and one mutation in the; R306C. R106W, T158M, and R270X are associated with severe clinical symptoms, whereas R306C is associated with less severe symptoms (5). Full-length MeCP2 variants were expressed in *Escherichia coli* BL21 CodonPlus (RIPL) cells transformed with a modified pTXB1-derived (New England Biolabs #N6707) construct encoding an N-terminal His_6_-SUMO tag and a C-terminal intein–chitin-binding domain. Transformed cells were grown at 37 °C in LB medium to an optical density at 600 nm (OD_600_) of approximately 0.6 to 0.7, cooled on ice for 30 to 45 min, and induced with 0.4 mM IPTG. Cultures were then incubated overnight at 16 °C. Cells from 1 L cultures were harvested by centrifugation and resuspended in 50 ml lysis buffer (buffer B2: 20 mM Na-Hepes, pH 7.0, 500 mM NaCl, 1 mM EDTA, and 0.2 mM AEBSF). To reduce viscosity and remove nucleic acid contamination, DNase I (5–10 μg mL^−1^) was added immediately prior to lysis. Cells were lysed by sonication with multiple 20 to 30 s pulses on a Bioruptor plus sonicator (Diagenode, B01020001), interspersed with cooling on ice. Lysates were clarified by centrifugation at 27,000×*g* for 25 min at 4 °C. The clarified supernatant was applied to 10 ml chitin affinity resin columns (New England Biolabs) pre-equilibrated with 10 column volumes of buffer B2, and the columns were incubated for 1 to 2 h at 4 °C with gentle mixing. The resin was washed with 3 column volumes of buffer B2 followed by 3 column volumes of cleavage buffer (buffer B3: 20 mM Na-Hepes, pH 8.5, 500 mM NaCl, 50 mM DTT, and 1 mM EDTA). On-column intein-mediated self-cleavage was induced by incubating the resin overnight at 4 °C in Buffer B3, thereby releasing the fusion protein lacking the C-terminal intein–chitin-binding domain tag. Cleaved protein was eluted with buffer B3 by gravity flow, and fractions were analyzed by SDS-PAGE. Fractions containing MeCP2 were pooled and concentrated using a 30 kDa Amicon centrifugal concentrator (Millipore Sigma, UFC810024). The pooled protein was then dialyzed overnight at 4 °C against 1 L of SUMO protease buffer (50 mM Hepes, pH 8.0, 150 mM NaCl, and 0.2% NP-40) containing SUMO protease. The N-terminal His_6_-SUMO tag was removed during this incubation. Following cleavage, the sample was incubated with Ni^2+^-affinity resin (Bio-Rad, 156012, 10 ml) pre-equilibrated in SUMO protease buffer for 2 h at 4 °C to remove the cleaved His_6_-SUMO tag and the His-tagged SUMO protease. Under these post-cleavage conditions, MeCP2 did not appreciably bind to the Ni^2+^ resin and was recovered predominantly in the flow-through, whereas the cleaved His_6_-SUMO fragment and His-tagged SUMO protease were retained on the resin. Minimal nonspecific retention of MeCP2 was confirmed by SDS-PAGE analysis of wash fractions. The flow-through containing tag-free MeCP2 was then applied to a 5 ml HiTrap Heparin HP column for further purification. Bound protein was eluted using a salt gradient from buffer A (50 mM Tris, pH 7.5, 100 mM NaCl, 0.1 mM EDTA, 0.1 mM TCEP) to buffer B (50 mM Tris, pH 7.5, 1 M NaCl, 0.1 mM EDTA, 0.1 mM TCEP). Fractions were analyzed by SDS-PAGE, and those containing purified MeCP2 were pooled and concentrated to approximately 1 mg mL^−1^ using a 30 kDa Amicon centrifugal concentrator. Purified protein was buffer-exchanged into the final storage buffer (25 mM Hepes, pH 7.5, 150 mM NaCl, and 10% glycerol), aliquoted, flash-frozen in liquid nitrogen, and stored at −80 °C until further use (Fig. S1).

### Mass spectrometry

SDS-PAGE gel bands containing full-length MeCP2 proteins (from above) were digested with a Tryptic Digestion Kit (Thermo Fisher Scientific #89871) according to the manufacturer’s protocol. Briefly, excised gel bands were destained with 200 μl of de-staining solution and then reduced in 30 μl of Reducing Agent Solution containing TCEP at 60 °C for 10 min with shaking at 700 rpm. The gel band was then treated with 30 μl of alkylating agent solution containing TCEP at room temperature in the dark for 60 min with shaking at 700 rpm. The entrained proteins were digested overnight in 25 μl of trypsin solution at 30 °C with shaking at 700 rpm. Finally, supernatants were recovered, dried with a SpeedVac, and resuspended in 10 μl of 0.1% TFA (in H_2_O) for LC-MS/MS analysis.

Each digested protein sample (3 μl) was analyzed on an Orbitrap Eclipse (Thermo Fisher Scientific) attached to a Vanquish Neo nano UPLC (Thermo Fisher Scientific). Peptides were separated with a nano HPLC column (75 μm × 20 cm, 1.7 μm C18; CoAnn Technologies #HEB07502001718IWF) in buffer A (0.1% formic acid in water). Peptides were eluted with a linear gradient from 3% to 25% of buffer B (0.1% formic acid in 80% acetonitrile) over 22 min. This was followed by increasing buffer B to 80% over 4 min and washing with 95% buffer B for 4 min. A FAIMS Pro source (Thermo Fisher Scientific) was located between the nanoESI source and the mass spectrometer. A selected compensation voltage was applied (−40V, −55V, and −75V) throughout the LC-MS/MS runs. One full MS scan was collected over a scan range of 350 to 1200 *m*/*z,* with a resolution of 120 K. Then, a series of MS2 scans were acquired for the most abundant ions from the MS1 scan using an ion trap analyzer. Ions were filtered with a charge of 2 to 5. An isolation window of 1.6 *m*/*z* was used in quadruple isolation mode. Ions were fragmented using high-energy collisional dissociation with collision energy of 30%.

Raw spectra were processed with Proteome Discoverer 3.0 software (Thermo Fisher Scientific). Database search criteria were: protein database; Methyl-CpG-binding Proteins 2 (Uniprot ID: P51608) and its mutation sequences; carboxyamidomethylated (+57 Da) at cysteine residues for fixed modifications; oxidized at methionine (+16 Da) residues; methionine-loss (−131.040 Da); methionine-loss + acetylation (−89.030 Da) at protein N-terminus for variable modifications; two maximum allowed missed cleavage; 10 ppm MS tolerance; and a 0.6-Da MS/MS tolerance. Only peptides resulting from trypsin digestion were considered, with a precursor MS tolerance of 10 ppm. The target-decoy approach was used to filter the search results, and the false discovery rate was < 1% at both the peptide and protein levels. For verification, parallel reaction monitoring was performed on tryptic peptides containing mutated residues. Resulting MS2 spectra were manually matched with theoretical MS2 spectra (Fig. S2), with protein sequence coverage maps shown in Figs. S3–S7.

### Nucleic acid targets

All short DNA and RNA oligonucleotides were purchased from Integrated DNA Technologies. Single-stranded DNA and RNA oligonucleotides for fluorescence polarization experiments were synthesized and 5′-labeled with 6-carboxy-fluorescein amidite and converted to double-stranded products by annealing equimolar amounts of complementary strands in annealing buffer (10 mM Tris-HCl, pH 7.5, 50 mM NaCl), heating to 95 °C for 5 min, and slow cooling to room temperature (Fig. S8). DNA and RNA sequences are shown in the respective data tables. One of the 26-mer RNAs (sequence 27) was derived from a prior MeCP2 SELEX experiment (44), and two additional 26-mer RNAs were designed with increased guanine content or reduced guanine and increased adenine content, respectively (sequences 31 and 32). RNA oligonucleotides were HPLC-purified. RNA secondary structures were predicted using the University of Rochester Medical Center RNAstructure (65) web server, and DNA secondary structures were predicted with the IDT OligoAnalyzer or OligoCalc (http://oligocalc.eu/).

The NPS plasmid containing the 147-bp nucleosome assembly 601 sequence (66) was used as a PCR template to create longer DNA templates and was a generous gift from the Poirier lab at Ohio State University. PCR primers were designed so that each amplicon included 24 bp of plasmid ‘linker’ DNA on each side of the 147 bp positioning sequence. Primer sequences were 5′-TCAACTCACTGCCCTATGC (forward primer) and 5′-GGAGGACACTGGGACATG (reverse primer), with the amplicon sequence and modified bases shown in Fig. S9.

Amplification reactions were assembled in a 96-well plate for each primer pair, with each PCR containing final concentrations of 25 ng plasmid DNA, 1.5 mM MgCl_2_, 0.2 mM dNTP, 0.2 μM of each primer, 1 mM DMSO, 1x Taq Buffer, and 0.5 units Taq polymerase (Syd Labs#MB118) in a 50 μl total reaction volume. Target DNA was amplified with an initial denaturation at 95 °C for 3 min, followed by 25 cycles of [95 °C for 45 s; 57 °C for 30 s; 72 °C for 90 s], and a final extension at 72 °C for 4 min. Similar reactions were pooled, concentrated, and buffer-exchanged into 1x TE buffer by centrifugation at 3000×*g* using a 100 KDa Amicon Column (Millipore-Sigma #UFC910008). DNA was concentrated and then run on a 10% polyacrylamide gel in 1x TBE at 200V for 4 h. The gel was stained with ethidium bromide, and the 195-bp band was excised from the gel. Gel pieces were crushed in a syringe and rotated overnight at room temperature in 1x TE. Residual polyacrylamide was removed by extraction with butanol and diethyl ether, and the DNA was dialyzed overnight against TE buffer. The concentration of purified DNA was then quantified using a NanoDrop 2000. Purified dsDNA was 5′-end labeled with gamma-^32^P as described by Maxam and Gilbert (67), and separated from free ^32^P on 15% native polyacrylamide native gels. Labeled target was recovered by the crush-and-soak method, concentrated with anhydrous n-butanol, and residual acrylamide removed by dialysis against 1× TE at 4 °C.

### dsDNA methylation

To create a fully methylated DNA target, we took 1 μg of 195 bp DNA from above and combined it into a 50 μl reaction containing 1 μl Msp1 methyltransferase (New England Biolabs #M0215S), 5.0 μl freshly prepared s-adenosylmethionine (3.2 mM final concentration), and 1x MspI reaction buffer. The reaction mixture was incubated overnight at 37 °C. The methylated DNA was purified with a Qiagen QIAquick PCR purification kit (Qiagen #28104) and eluted with 20 μl of 1x TE buffer. We verified DNA methylation by digesting 2 μl of methylated DNA with either MspI or HpaII restriction enzymes. Any visible HpaII digestion was taken as evidence that the methylation reaction was incomplete. We also used 500 ng of methylated DNA as input for the Zymo EZ DNA Methylation Kit (#D5001). After bisulfite conversion and clean-up, the resulting DNA was end-repaired (EpiCentre #ER0720), subcloned into a pUC19 plasmid with a blunt/TA master mix (New England Biolabs #M0367S), transformed into *E. coli*, and sequenced at the University of Chicago Comprehensive Cancer Center DNA Sequencing Facility. We then used BiQ Analyzer software (68) to align and designate converted cytosine residues. 10 clones were sequenced, and all 10 clones showed complete methylation at the target CpG sites.

### Fluorescence polarization assays

Fluorescence polarization was used in most experiments to monitor MeCP2–nucleic acid interactions (69). Binding reactions contained purified WT or mutant MeCP2 proteins serially diluted from a high starting concentration in the presence of a fixed concentration (1–5 nM) of fluorescein amidite-labeled nucleic acid in binding buffer (25 mM Hepes, pH 8.0, 150 mM NaCl, 1 mM TCEP, 10% [v/v] glycerol). Reactions were assembled in 384-well black assay plates (Corning, #3575), incubated for 20 to 30 min at room temperature, and measured using a Synergy Neo2 multi-mode microplate reader (BioTek) with excitation and emission wavelengths of 485 and 530 nm, respectively. Polarization values (P) were converted to anisotropy (A) using the equation (A) = (2 × P)/(3 – P) (70). Background values from negative control wells containing labeled DNA or RNA in binding buffer alone were subtracted before analysis. Background-corrected anisotropy values were then plotted as a function of total protein concentration and fit by nonlinear regression in KaleidaGraph 4.0 using the Hill equation, *Y = B*_*max*_ *× X*^*h*^ */ (K*_*d*_^*h*^ *+ X*^*h*^*)*, where *Y* is the observed signal, *B*_*max*_ is the maximal binding signal, *X* is the total protein concentration, *h* is the Hill slope, and *K*_*d*_ is the apparent midpoint of the binding curve. Because this formula uses total protein concentration and does not explicitly account for ligand depletion, the fitted *K*_*d*_ and *h* values should only be considered apparent parameters for comparative interpretation.

The DNA concentration (1–5 nM) approached the lower range of measured affinities in some experiments, so data were also fit using a quadratic binding equation that accounts for ligand depletion (modified from (71) for anisotropy fit): $Y=r_{f}+\left(r_{b}-r_{f}\right)\left[\left(R_{T}+L_{T}+K_{d}-\sqrt{{\left(R_{T}+L_{T}+K_{d}\right)}^{2}-4R_{T}L_{T}}\right)/\left(2L_{T}\right)\right]$, where $R_{T}$ is total protein concentration, $L_{T}$ is total fluorescent ligand concentration, and $r_{f}$ and $r_{b}$ are the anisotropies of free and bound ligand, respectively. Both fitting calculations yielded similar apparent *K*_*d*_ values, consistent with the low ligand concentration relative to most of the titration range. Data for both calculations are reported as mean ± SD from at least three independent experiments.

### Electrophoretic mobility shift assays

MeCP2 binding reactions to the 195 bp fragment were carried out in 25 mM Hepes (pH 8.0), 150 mM NaCl, 10% (v/v) glycerol, 0.05% (v/v) Tween 20, 1 mM TCEP, and 0.1 mg mL^−1^ bovine serum albumin at 20 ± 1 °C for 30 min using 2 nM DNA and 0 to 30 nM WT MeCP2; 0 to 19 nM R270X; 0 to 31 nM R306C; 0 to 33 nM T158M; and 0 to 40 nM R106W. Each EMSA experiment was performed at least twice. Products were analyzed on 10 to 12% polyacrylamide gels (37.5:1 acrylamide: bis-acrylamide) in 1x TBE running buffer. After electrophoresis, autoradiographic images were imprinted on storage phosphor screens (GE Healthcare) and then detected with an Amersham Typhoon laser scanner. Bands were quantified with the Image-Quant TL software (GE Healthcare) as described by the manufacturer.

To calculate the binding of *n* protein molecules (*P*) to a single DNA (*nP* + *D* ⇆ *P*_*n*_*D*), the association constant is *K*_*n*_ = [*P*_*n*_*D*]/[*P*]^*n*^[*D*]. Separating variables and taking natural logarithms gives the linear relationship ln(*[P*_*n*_*D]/[D])* = *n* ln[*P*] + ln *K*_*n*_. The slope of the graph represents the stoichiometry *n*, while the titration midpoint is the formation constant *K*_*n*_ (where ln*[P*_*n*_*D]/[D]* = 0, ln *K*_*n*_ = -*n* ln[*P*]). The free protein concentration for each titration step [*P*] was calculated using the conservation relation *[P] = [P]*_*tot*_ *– n[P*_*n*_*D]*, in which *[P]*_*tot*_ is the total protein concentration and *n* is an initial estimate of the stoichiometry. An updated estimate for *n* was then obtained from the graph of the linear dependence of ln*[P*_*n*_*D]/[D]* on ln*[P].* The newly obtained value of *n* was then used in the conservation relation, and the cycle was iterated until the values of *n* stopped changing. Values of *K*_*n*_ are difficult to compare when complexes differ in stoichiometry. However, the assumption of equipartition of binding free energies allows us to evaluate monomer-equivalent association constants (*K*_*mono*_ *= (K*_*n*_*)*^*1/n*^), which are easier to compare (72).

## Data availability

All data are included in the manuscript and supporting information.

## Supporting information

This article contains supporting information (71).

## Conflict of interest

The authors declare that they have no conflicts of interest with the contents of this article.
