# Supplementary material for: DNA methylation, nucleic acid structure, and rett mutations tune MeCP2 binding affinity and cooperativity
Source: J Biol Chem. 2026 Jun 4;302(7):113227. doi: 10.1016/j.jbc.2026.113227 (PMC13333338; doi:10.1016/j.jbc.2026.113227)
Supplement: Supporting Tables [file mmc1.docx]

**Table S1. Binding affinity (*K_d_)* for MeCP2 variants and single-stranded oligonucleotide targets**, calculated from Eq [8] in Pollard, 2010. Binding affinities (*K_d_*) are in nM. Data were fitted to the quadratic equation and represent mean ± s.d. from three independent experiments. Base modifications mentioned in the text are in bold. Potential secondary structures are shown in **Fig S10**.

| Name | ID | Sequence | | **WT** | **R270X** | **R306C** | **T158M** | **R106W** |
| --- | --- | --- | --- | --- | --- | --- | --- | --- |
| 18ss-UM | 1 | | FAM–5’–GTTGCCGCGTGGTGGCAG | *K_d_* = 8.8 ± 0.7 | *K_d_* = 3.9 ± 0.3 | *K_d_* = 8.1 ± 0.4 | *K_d_* = 42.4 ± 3.3 | *K_d_* = 141 ± 10 |
| 18ss-1M | 2 | | FAM–5’–GTTGCCGCGTGGTG**C**CAG | *K_d_* = 8.2 ± 0.5 | *K_d_* = 4.9 ± 0.4 | *K_d_* = 7.2 ± 0.4 | *K_d_* = 20.6 ± 1.8 | *K_d_* = 39.8 ± 3.6 |
| 18ss-2M | 3 | | FAM–5’–GTTGCCGC**C**TGGTG**C**CAG | *K_d_* = 16.6 ± 1.2 | *K_d_* = 7.4 ± 0.6 | *K_d_* = 16.9 ± 1.6 | *K_d_* = 28.0 ± 1.8 | *K_d_* = 66.6 ± 4.6 |
|  |  | |  |  |  |  |  |  |
| 19ss-UM | 4 | | FAM–5’–AGGCCGCCAGAGAGCGCCC | *K_d_* = 86.7 ± 8.2 | *K_d_* = 43.4 ± 4.5 | *K_d_* = 105.7 ± 8.4 | *K_d_* = 189 ± 17 | *K_d_* = 429 ± 28 |
|  |  | |  |  |  |  |  |  |
| 25ss-UM | 5 | | FAM–5’–GCTAGGCCGCCAGAGAGCGCCCAAC | *K_d_* = 8.2 ± 0.7 | *K_d_* = 5.2 ± 0.3 | *K_d_* = 8.9 ± 1.0 | *K_d_* = 17.6 ± 1.7 | *K_d_* = 40.9 ± 3.5 |
| 25ss-1M | 6 | | FAM–5’–GCTAGGCCGCC**G**GAGAGCGCCCAAC | *K_d_* = 5.8 ± 0.8 | *K_d_* = 4.0 ± 0.4 | *K_d_* = 13.2 ± 1.4 | *K_d_* = 17.5 ± 1.2 | *K_d_* = 46.2 ± 3.3 |

**Table S2. Binding affinities for MeCP2 variants, single-stranded DNA substrates, and their corresponding dsDNAs**, calculated from Eq [8] in Pollard, 2010. Binding affinities (*K_d_*) are in nM. Data were fitted to the quadratic equation and represent mean ± s.d. from three independent experiments. Some ssDNA data from **Table S1** are repeated here to facilitate side-by-side comparisons.

| Name | ID | Sequence | | **WT** | | **R270X** | | **R306C** | | **T158M** | | **R106W** | |
| --- | --- | --- | --- | --- | --- | --- | --- | --- | --- | --- | --- | --- | --- |
| 18ss-UM | 1 | FAM–5’–GTTGCCGCGTGGTGGCAG | *K_d_* = 8.8 ± 0.7 | | *K_d_* = 3.9 ± 0.3 | | *K_d_* = 8.1 ± 0.4 | | *K_d_* = 42.4 ± 3.3 | | *K_d_* = 141 ± 10 | |  |
| 18ds-UM | 7 | FAM–5’–GTTGCCGCGTGGTGGCAG  CAACGGCGCACCACCGTC-5’ | *K_d_* = 7.4 ± 0.8 | | *K_d_* = 6.0 ± 0.6 | | *K_d_* = 6.8 ± 0.9 | | *K_d_* = 6.4 ± 0.6 | | *K_d_* = 24.4 ± 2.0 | |  |
|  |  |  |  | |  | |  | |  | |  | |  |
| 19ss-UM | 4 | FAM–5’–AGGCCGCCAGAGAGCGCCC | *K_d_* = 86.7 ± 8.2 | | *K_d_* = 43.4 ± 4.5 | | *K_d_* = 105.7 ± 8.4 | | *K_d_* = 189 ± 17 | | *K_d_* = 429 ± 28 | |  |
| 19ds-UM | 8 | FAM–5’–AGGCCGCCAGAGAGCGCCC  TCCGGCGGTCTCTCGCGGG-5’ | *K_d_* = 6.4 ± 0.6 | | *K_d_* = 6.1 ± 0.8 | | *K_d_* = 6.5 ± 0.7 | | *K_d_* = 13.5 ± 1.0 | | *K_d_* = 32.0 ± 2.9 | |  |
|  |  |  |  | |  | |  | |  | |  | |  |
| 25ss-UM | 5 | FAM–5’–GCTAGGCCGCCAGAGAGCGCCCAAC | *K_d_* = 8.2 ± 0.7 | | *K_d_* = 5.2 ± 0.3 | | *K_d_* = 8.9 ± 1.0 | | *K_d_* = 17.6 ± 1.7 | | *K_d_* = 40.9 ± 3.5 | |  |
| 25ds-UM | 10 | FAM–5’–GCTAGGCCGCCAGAGAGCGCCCAAC  CGATCCGGCGGTCTCTCGCGGGTTG | *K_d_* = 3.8 ± 0.5 | | *K_d_* = 3.5 ± 0.4 | | *K_d_* = 3.4 ± 0.4 | | *K_d_* = 5.6 ± 0.7 | | *K_d_* = 8.9 ± 0.8 | |  |

**Table S3. Binding affinity (*K_d_)* for MeCP2 variants and methylated, single-stranded DNA targets**, calculated from Eq [8] in Pollard, 2010. Binding affinities (*K_d_*) are in nM. Data were fitted to the quadratic equation and represent mean ± s.d. from three independent experiments. Methylated bases (5mC) within the canonical CpG dyad are underlined. Some ssDNA data from **Table S1** are repeated here to facilitate side-by-side comparisons.

| Name | ID | Sequence | | **WT** | | **R270X** | | **R306C** | | **T158M** | | **R106W** | |
| --- | --- | --- | --- | --- | --- | --- | --- | --- | --- | --- | --- | --- | --- |
| 18ss-UM | 1 | FAM–5’–GTTGCCGCGTGGTGGCAG | *K_d_* = 8.8 ± 0.7 | | *K_d_* = 3.9 ± 0.3 | | *K_d_* = 8.1 ± 0.4 | | *K_d_* = 42.4 ± 3.3 | | *K_d_* = 141 ± 10 | |  |
| 18ss-SM | 11 | FAM–5’–GTTGC**C**GCGTGGTGGCAG | *K_d_* = 4.3 ± 0.5 | | *K_d_* = 1.3 ± 0. 2 | | *K_d_* = 3.6 ± 0.4 | | *K_d_* = 5.5 ± 0.4 | | *K_d_* = 22.6 ± 2.9 | |  |
| 18ss-DM-C | 12 | FAM–5’–GTTGC**C**G**C**GTGGTGGCAG | *K_d_* = 4.6 ± 0.6 | | *K_d_* = 1.7 ± 0.2 | | *K_d_* = 4.4 ± 0.5 | | *K_d_* = 7.4 ± 0.6 | | *K_d_* = 23.1 ± 2.3 | |  |
|  |  |  |  | |  | |  | |  | |  | |  |
| 19ss-UM | 4 | FAM–5’–AGGCCGCCAGAGAGCGCCC | *K_d_* = 86.5 ± 8.5 | | *K_d_* = 43.4 ± 4.5 | | *K_d_* = 105.7 ± 8.4 | | *K_d_* = 189 ± 17 | | *K_d_* = 429 ± 28 | |  |
| 19ss-SM | 13 | FAM–5’–AGGC**C**GCCAGAGAGCGCCC | *K_d_* = 27.2 ± 1.9 | | *K_d_* = 13.9± 1.1 | | *K_d_* = 27.2 ± 1.8 | | *K_d_* = 91.6 ± 7.5 | | *K_d_* = 257 ± 23 | |  |
| 19ss-DM-S | 14 | FAM–5’–AGGC**C**GCCAGAGAG**C**GCCC | *K_d_* = 15.4 ± 1.6 | | *K_d_* = 6.9 ± 0.7 | | *K_d_* = 18.8 ± 2.2 | | *K_d_* = 26.4 ± 1.5 | | *K_d_* = 123.7 ± 8.3 | |  |
|  |  |  |  | |  | |  | |  | |  | |  |
| 25ss-UM | 5 | FAM–5’–GCTAGGCCGCCAGAGAGCGCCCAAC | *K_d_* = 8.2 ± 0.7 | | *K_d_* = 5.2 ± 0.3 | | *K_d_* = 8.9 ± 1.0 | | *K_d_* = 17.6 ± 1.7 | | *K_d_* = 40.9 ± 3.5 | |  |
| 25ss-1M | 6 | FAM–5’–GCTAGGCCGCC**G**GAGAGCGCCCAAC | *K_d_* = 5.8 ± 0.8 | | *K_d_* = 4.0 ± 0.4 | | *K_d_* = 13.2 ± 1.4 | | *K_d_* = 17.5 ± 1.2 | | *K_d_* = 46.2 ± 3.3 | |  |
| 25ss-DM-S | 15 | FAM–5’–GCTAGGC**C**GCCAGAGAG**C**GCCCAAC | *K_d_* = 2.9 ± 0.4 | | *K_d_* = 1.3 ± 0.1 | | *K_d_* = 2.5 ± 0.3 | | *K_d_* = 2.8 ± 0.2 | | *K_d_* = 8.7 ± 0.7 | |  |
| 25ss-TM-C2S1 | 16 | FAM–5’–GCTAGGC**C**GC**C**GGAGAG**C**GCCCAAC | *K_d_* = 4.1 ± 0.4 | | *K_d_* = 2.1 ± 0.2 | | *K_d_* = 2.1 ± 0.3 | | *K_d_* = 4.8 ± 0.4 | | *K_d_* = 12.6 ± 1.2 | |  |

**Table S4**. **Binding affinity (*K_d_)* for MeCP2 variants and double-stranded oligonucleotide targets,** , calculated from Eq [8] in Pollard, 2010. Binding affinities (*K_d_*) are in nM. Data were fitted to the quadratic equation and represent mean ± s.d. from three independent experiments. Methylated bases (5mC) within the canonical CpG dyad are underlined. Some dsDNA data from **Table S2** are repeated here to facilitate side-by-side comparisons.

| Name | ID | Sequence | | **WT** | | **R270X** | | **R306C** | | **T158M** | | **R106W** | |
| --- | --- | --- | --- | --- | --- | --- | --- | --- | --- | --- | --- | --- | --- |
| 18ds-UM | 7 | FAM–5’–GTTGCCGCGTGGTGGCAG  CAACGGCGCACCACCGTC-5’ | *K_d_* = 7.4 ± 0.8 | | *K_d_* = 6.0 ± 0.6 | | *K_d_* = 6.8 ± 0.9 | | *K_d_* = 6.4 ± 0.6 | | *K_d_* = 24.4 ± 2.0 | |  |
| 18ds-SM | 17 | FAM–5’–GTTGC**C**GCGTGGTGGCAG  CAACGGCGCACCACCGTC-5’ | *K_d_* = 5.2 ± 0.8 | | *K_d_* = 2.9 ± 0.3 | | *K_d_* = 4.5 ± 0.5 | | *K_d_* = 3.6 ± 0.2 | | *K_d_* = 18.4 ± 1.1 | |  |
| 18ds-Rev-SM | 18 | FAM–5’–GTTGCCGCGTGGTGGCAG  CAACGG**C**GCACCACCGTC-5’ | *K_d_* = 8.8 ± 1.0 | | *K_d_* = 6.5 ± 0.7 | | *K_d_* = 7.1 ± 0.6 | | *K_d_* = 6.4 ± 0.5 | | *K_d_* = 23.7 ± 2.0 | |  |
| 18ds-FM | 19 | FAM–5’–GTTGC**C**GCGTGGTGGCAG  CAACGG**C**GCACCACCGTC-5’ | *K_d_* = 5.0 ± 0.8 | | *K_d_* = 3.1 ± 0.3 | | *K_d_* = 5.9 ± 0.5 | | *K_d_* = 3.2 ± 0.2 | | *K_d_* = 8.0 ± 0.6 | |  |
| 18ds-DM-C | 20 | FAM–5’–GTTGC**C**G**C**GTGGTGGCAG  CAACGGCGCACCACCGTC-5’ | *K_d_* = 3.8 ± 0.5 | | *K_d_* = 2.6 ± 0.3 | | *K_d_* = 3.9 ± 0.3 | | *K_d_* = 3.8 ± 0.3 | | *K_d_* = 18.4 ± 1.9 | |  |
|  |  |  |  | |  | |  | |  | |  | |  |
| 19ds-UM | 8 | FAM–5’–AGGCCGCCAGAGAGCGCCC  TCCGGCGGTCTCTCGCGGG-5’ | *K_d_* = 6.4 ± 0.6 | | *K_d_* = 6.1 ± 0.8 | | *K_d_* = 6.5 ± 0.7 | | *K_d_* = 13.5 ± 1.0 | | *K_d_* = 32.0 ± 2.9 | |  |
| 19ds-SM | 21 | FAM–5’–AGGC**C**GCCAGAGAGCGCCC  TCCGGCGGTCTCTCGCGGG-5’ | *K_d_* = 4.5 ± 0.6 | | *K_d_* = 3.6 ± 0.4 | | *K_d_* = 5.6 ± 0.7 | | *K_d_* = 7.1 ± 0.3 | | *K_d_* = 21.7 ± 1.9 | |  |
| 19ds-Rev-SM | 22 | FAM–5’–AGGCCGCCAGAGAGCGCCC  TCCGG**C**GGTCTCTCGCGGG-5’ | *K_d_* = 7.3 ± 0.8 | | *K_d_* = 7.4 ± 0.6 | | *K_d_* = 6.8 ± 0.8 | | *K_d_* =11.1 ±1.0 | | *K_d_* = 27.0 ± 2.1 | |  |
| 19ds-DM-S | 23 | FAM–5’–AGGC**C**GCCAGAGAG**C**GCCC  TCCGGCGGTCTCTCGCGGG-5’ | *K_d_* = 19.1 ± 2.2 | | *K_d_* = 10.3 ± 08 | | *K_d_* = 16.1 ± 1.8 | | *K_d_* = 15.6 ± 0.8 | | *K_d_* = 63.6 ± 4.9 | |  |
|  |  |  |  | |  | |  | |  | |  | |  |
| 25ds-UM | 10 | FAM–5’–GCTAGGCCGCCAGAGAGCGCCCAAC  CGATCCGGCGGTCTCTCGCGGGTTG | *K_d_* = 3.8 ± 0.5 | | *K_d_* = 3.5 ± 0.4 | | *K_d_* = 3.4 ± 0.4 | | *K_d_* = 6.2 ± 0.7 | | *K_d_* = 8.9 ± 0.8 | |  |
| 25ds-1M | 24 | FAM–5’–GCTAGGCCGCCGGAGAGCGCCCAAC  CGATCCGGCGGCCTCTCGCGGGTTG | *K_d_* = 2.9 ± 0.4 | | *K_d_* = 2.9 ± 0.4 | | *K_d_* = 3.2 ± 0.4 | | *K_d_* = 4.0 ± 0.5 | | *K_d_* = 9.3 ± 1.0 | |  |
| 25ds-DM-S | 25 | FAM–5’–GCTAGGC**C**GCCAGAGAG**C**GCCCAAC  CGATCCGGCGGTCTCTCGCGGGTTG | *K_d_* = 2.2 ± 0.3 | | *K_d_* = 1.5 ± 0.2 | | *K_d_* = 2.4 ± 0.4 | | *K_d_* = 2.8 ± 0.3 | | *K_d_* = 5.0 ± 0.6 | |  |
| 25ds-TM-C2S1 | 26 | FAM–5’–GCTAGGC**C**GC**C**GGAGAG**C**GCCCAAC  CGATCCGGCGGTCTCTCGCGGGTTG | *K_d_* = 2.7 ± 0.3 | | *K_d_* = 2.6 ± 0.3 | | *K_d_* = 1.9 ± 0.3 | | *K_d_* = 3.1 ± 0.4 | | *K_d_* = 6.8 ± 0.7 | |  |

**Table S5. MeCP2 variant binding affinity for single- and double-stranded RNA and DNA targets**, calculated from Eq [8] in Pollard, 2010. Binding affinities (*K_d_*) are in nM. Data were fitted to the quadratic equation and represent mean ± s.d. from three independent experiments. Potential secondary structures are shown in **Fig S10**.

| Name | ID | Sequence | | **WT** | | **R270X** | | **R306C** | **T158M** | | | **R106W** |
| --- | --- | --- | --- | --- | --- | --- | --- | --- | --- | --- | --- | --- |
| RNA1 | 27 | FAM-5’-GCGGUGUAUAGCCUAAUCUUUACCGC | *K_d_* = 8.5 ± 0.6 | | *K_d_* = 7.5 ± 0.5 | | *K_d_* = 8.7 ± 0.7 | | | *K_d_* = 10.0 ± 0.8 | *K_d_* = 14.0 ± 0.9 | |
| DNA1 | 28 | FAM-5’-GCGGTGTATAGCCTAATCTTTACCGC | *K_d_* = 2.8 ± 0.3 | | *K_d_* = 2.5 ± 0.2 | | *K_d_* = 2.9 ± 0.2 | | | *K_d_* = 11.3 ± 0.6 | *K_d_* = 32.8 ± 2.1 | |
| dsRNA1 | 29 | FAM-5’-GCGGUGUAUAGCCUAAUCUUUACCGC  CGCCACAUAUCGGAUUAGAAAUGGCG-5’ | *K_d_* = 14.5 ± 1.7 | | *K_d_* = 8.3 ± 0.8 | | *K_d_* = 10.4 ± 0.7 | | | *K_d_* = 8.0 ± 1.0 | *K_d_* = 16.9 ± 1.2 | |
| dsDNA1 | 30 | FAM-5’-GCGGTGTATAGCCTAATCTTTACCGC  CGCCACATATCGGATTAGAAATGGCG-5’ | *K_d_* = 3.0 ± 0.3 | | *K_d_* = 3.9 ± 0.5 | | *K_d_* = 2.3 ± 0.3 | | | *K_d_* = 2.1 ± 0.2 | *K_d_* = 4.3 ± 0.3 | |
| RNA2 | 31 | FAM-5’-GCGGUGCGGUGCCUAAUGGGGACCGC | *K_d_* = 7.8 ± 0.6 | | *K_d_* = 5.7 ± 0.5 | | *K_d_* = 8.2 ± 0.6 | | | *K_d_* = 11.6 ± 1.1 | *K_d_* = 13.0 ± 1.1 | |
| RNA3 | 32 | FAM-5’-CGCAAAAACACAUAAAUCAAAACCAA | *K_d_* = 10.8 ± 0.6 | | *K_d_* = 8.7 ± 0.7 | | *K_d_* = 10.3± 0.7 | | | *K_d_* = 14.4 ± 0.9 | *K_d_* = 18.9 ± 1.3 | |
