# Supplementary material for: DNA methylation, nucleic acid structure, and rett mutations tune MeCP2 binding affinity and cooperativity
Source: J Biol Chem. 2026 Jun 4;302(7):113227. doi: 10.1016/j.jbc.2026.113227 (PMC13333338; doi:10.1016/j.jbc.2026.113227)
Supplement: Supporting Figures [file mmc2.docx]

**DNA methylation, nucleic acid structure, and Rett mutations tune MeCP2 binding affinity and cooperativity**

Manana Melikishvili^1^, Matthew Rea^1^, Colt Capan^3^, Lee Hyoungjoo^3^, Darrell P. Chandler^1^, and Yvonne Fondufe-Mittendorf^1,2^*

**Supplemental Figures**

**Fig S1**. Purified MeCP2 proteins used for mass spectrometry and gel shift assays.

**Fig S2**. Mass spectra verify the presence of expected MeCP2 mutations.

**Fig S3**. LC-MS/MS protein sequence coverage map for wildtype MeCP2.

**Fig S4**. LC-MS/MS protein sequence coverage map for MeCP2 variant R106W.

**Fig S5**. LC-MS/MS protein sequence coverage map for MeCP2 variant T158M.

**Fig S6**. LC-MS/MS protein sequence coverage map for MeCP2 variant R270X.

**Fig S7**. LC-MS/MS protein sequence coverage map for MeCP2 variant R306C.

**Fig S8**. FAM-labeled single- and double-stranded DNA and RNA oligonucleotides.

**Fig S9**. 195 bp target DNA sequence and methylated sites.

**Fig S10**. Potential secondary structures for single-stranded DNA and RNA oligonucleotides.

**Fig S11**. MeCP2 binding to an unmethylated and fully methylated 195-bp DNA.





**Fig S1. Purified MeCP2 proteins used for mass spectrometry and gel shift assays.** The calculated molecular weight of MECP2 is 52 KDa, and the post-translationally modified MeCP2 protein is ~75-80 KDa. Lanes 1 and 7 = molecular weight markers (in kDa); 2 = wildtype MeCP2; 3 = variant R306C; 4 = T158M; 5 = R106W; 6 = R270X.

**

**

**Fig S2. Mass spectra verify the presence of expected MeCP2 mutations.** Purified proteins from **Fig S1** were analyzed by LC-MS/MS, targeting the selected peptide in **A**. **B**, Fragmentation spectra for the selected peptide were manually annotated. The R270X mutation generates a truncated protein, so there is no *m/z* trace for a peptide fragment or amino acid substitution.


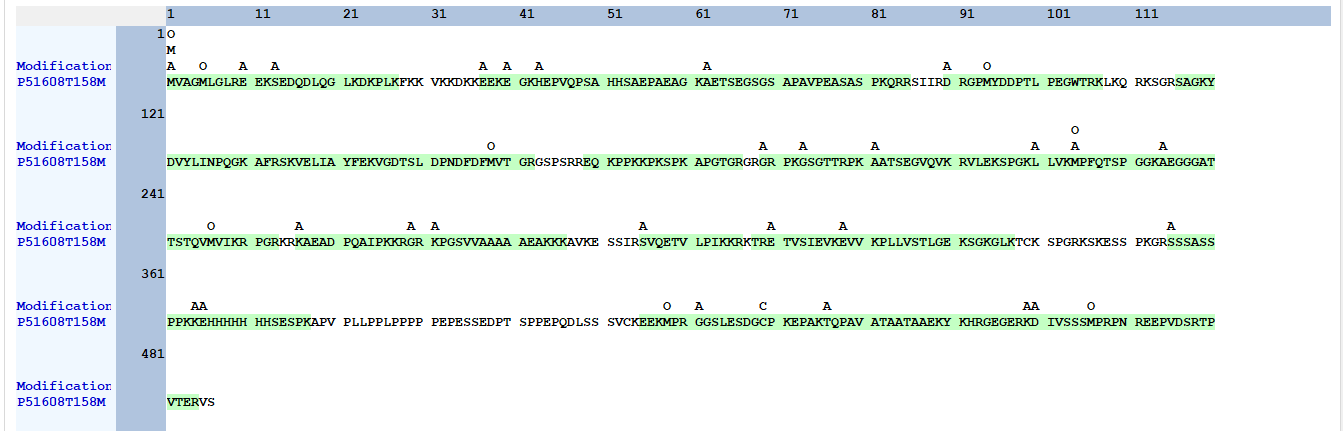


**Fig S3. LC-MS/MS protein sequence coverage map for wildtype MeCP2.** The total sequence coverage was 86% (green highlights). A = acetyl (N-term); M = methione loss (N-term); N = methione loss + acetylation (N-term); O = oxidation (M); C = carboxyamidomethylation (cystein).

**Fig S4. LC-MS/MS protein sequence coverage map for MeCP2 variant R106W.** The total sequence coverage was 74% (green highlights). A = acetyl (N-term); M = methione loss (N-term); N = methione loss + acetylation (N-term); O = oxidation (M); C = carboxyamidomethylation (cystein); * = mutated residue.

**Fig S5.** **LC-MS/MS protein sequence coverage map for MeCP2 variant T158M.** The total sequence coverage was 80% (green highlights). A = acetyl (N-term); M = methione loss (N-term); N = methione loss + acetylation (N-term); O = oxidation (M); C = carboxyamidomethylation (cystein); * = mutated residue.

**
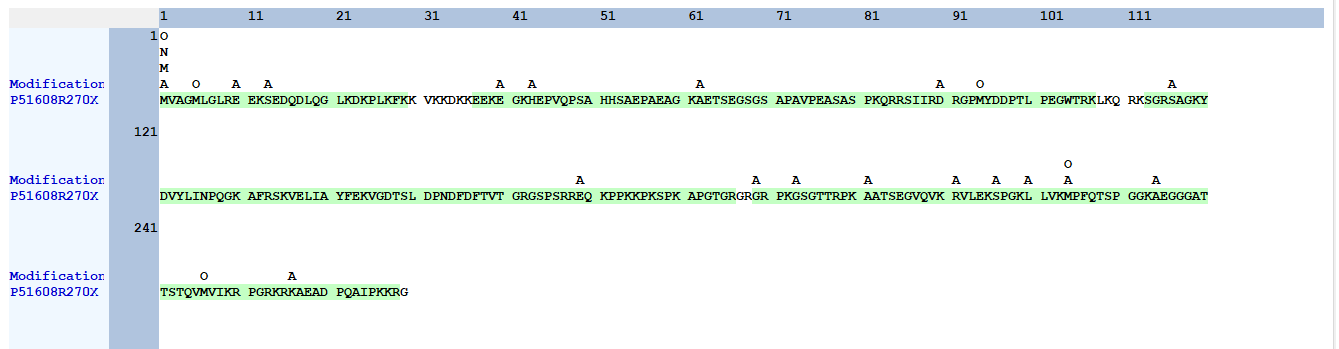
**

**Fig S6. LC-MS/MS protein sequence coverage map for MeCP2 variant R270X.** The total sequence coverage was 94% (green highlights). A = acetyl (N-term); M = methione loss (N-term); N = methione loss + acetylation (N-term); O = oxidation (M); C = carboxyamidomethylation (cystein). R270X is a truncated protein, so there is no mutated residue.

**Fig S7. LC-MS/MS protein sequence coverage map for MeCP2 variant R306C**. The total sequence coverage (green highlights) was 80%. A = acetyl (N-term); M = methione loss (N-term); N = methione loss + acetylation (N-term); O = oxidation (M); C = carboxyamidomethylation (cystein); * = mutated residue.





**Fig S8. FAM-labeled single- and double-stranded DNA and RNA oligonucleotides.** Oligonucleotides were separated on 4% NuSieve agarose gels in 1xTBE buffer. Oligonucleotides and their sequences are in **Tables 1-6**. M = 50 bp molecular weight marker.


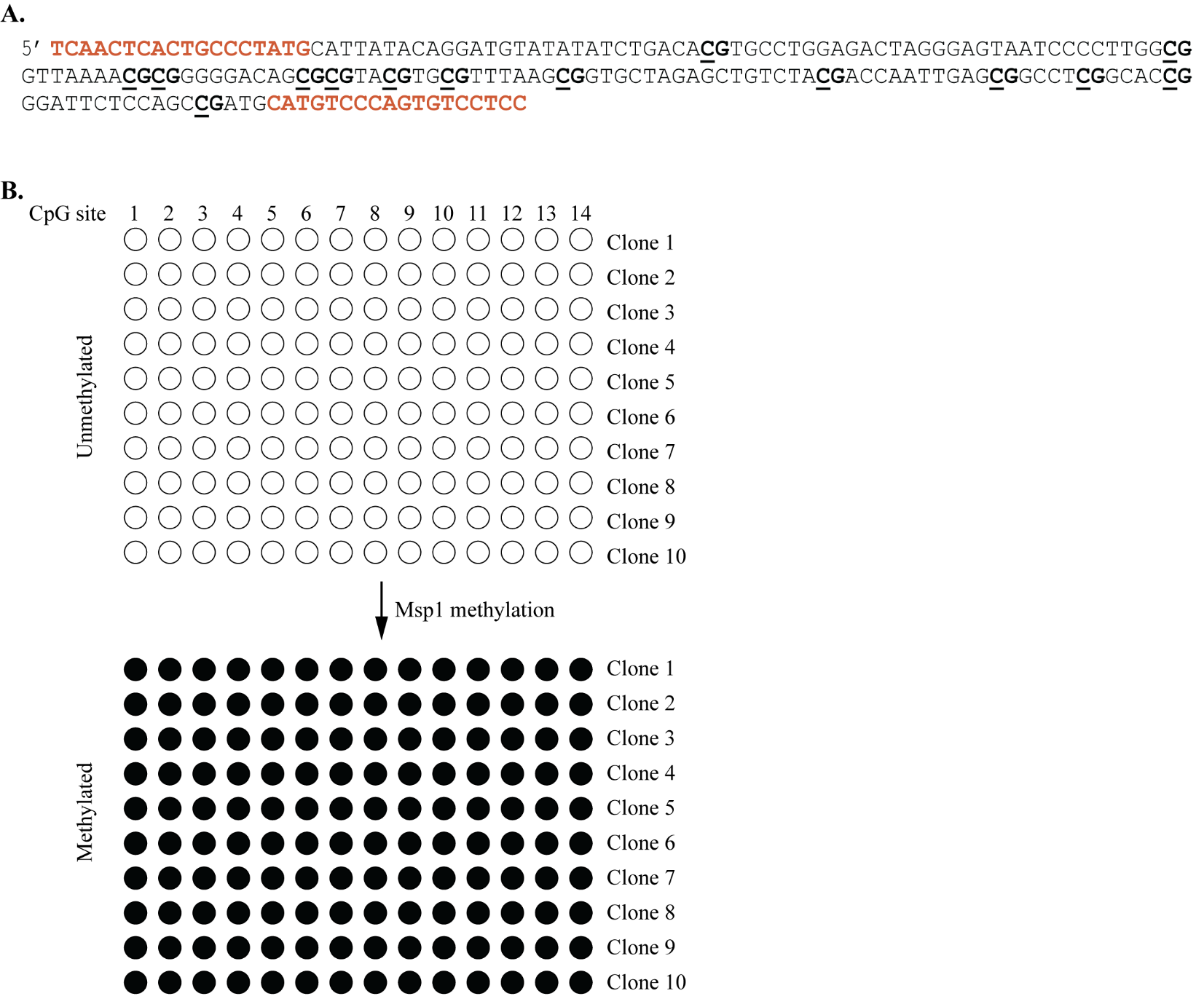


**Fig S9. 195 bp target DNA sequence and methylated sites**. **A**, CpG sites in the Widom 601 sequence are in bold font. Red letters are PCR primer sequences. **B,** Complete methylation was verified by bisulfite sequencing of 10 independent clones. After bisulfite conversion, unmethylated cytosines are read as thymine (C→T), whereas methylated cytosines are read as cytosine. In the lollipop plot, white circles are unmethylated cytosine, black circles are methylated cytosines.


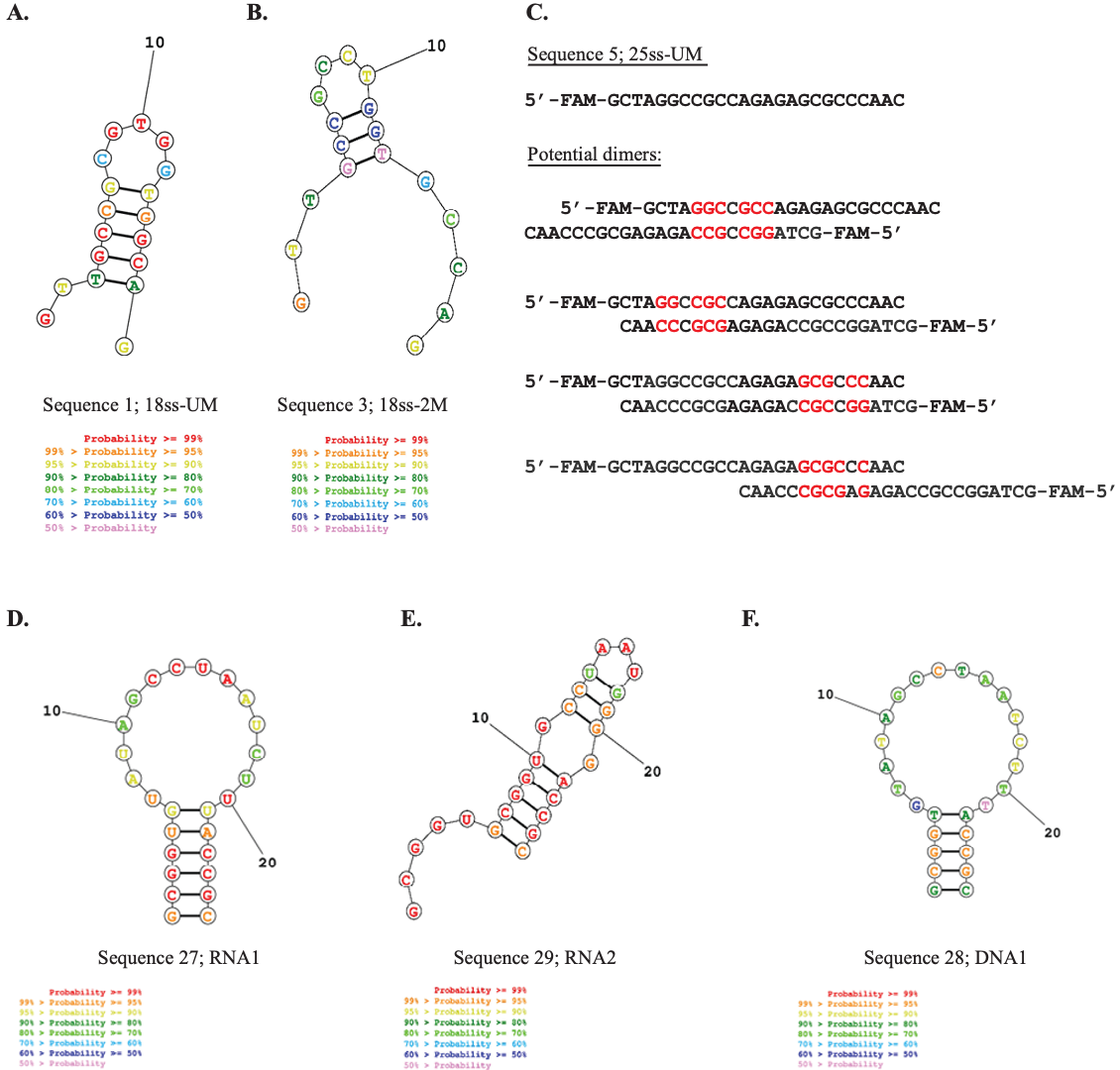


**Fig S10. Potential secondary structures for single-stranded DNA and RNA oligonucleotides**. DNA oligonucleotide self-complementarity and folding probabilities were determined using the IDT OligoAnalyzer or OligoCalc (http://oligocalc.eu/) software. RNA oligonucleotide self-complementarity and folding probabilities were determined using the University of Rochester Medical Center RNAstructure web server. **A**, the unmodified 18-mer ssDNA oligonucleotide. **B**, same as in **A**, except with two nucleotide substitutions. Note that the calculated probability of hairpin formation is <50%.**C**, the 25-mer ssDNA and potential dimers assuming a 1 bp mismatch. **D**, RNA1. **E**, RNA2, **F**, same sequence as **D**, except as a DNA oligonucleotide.


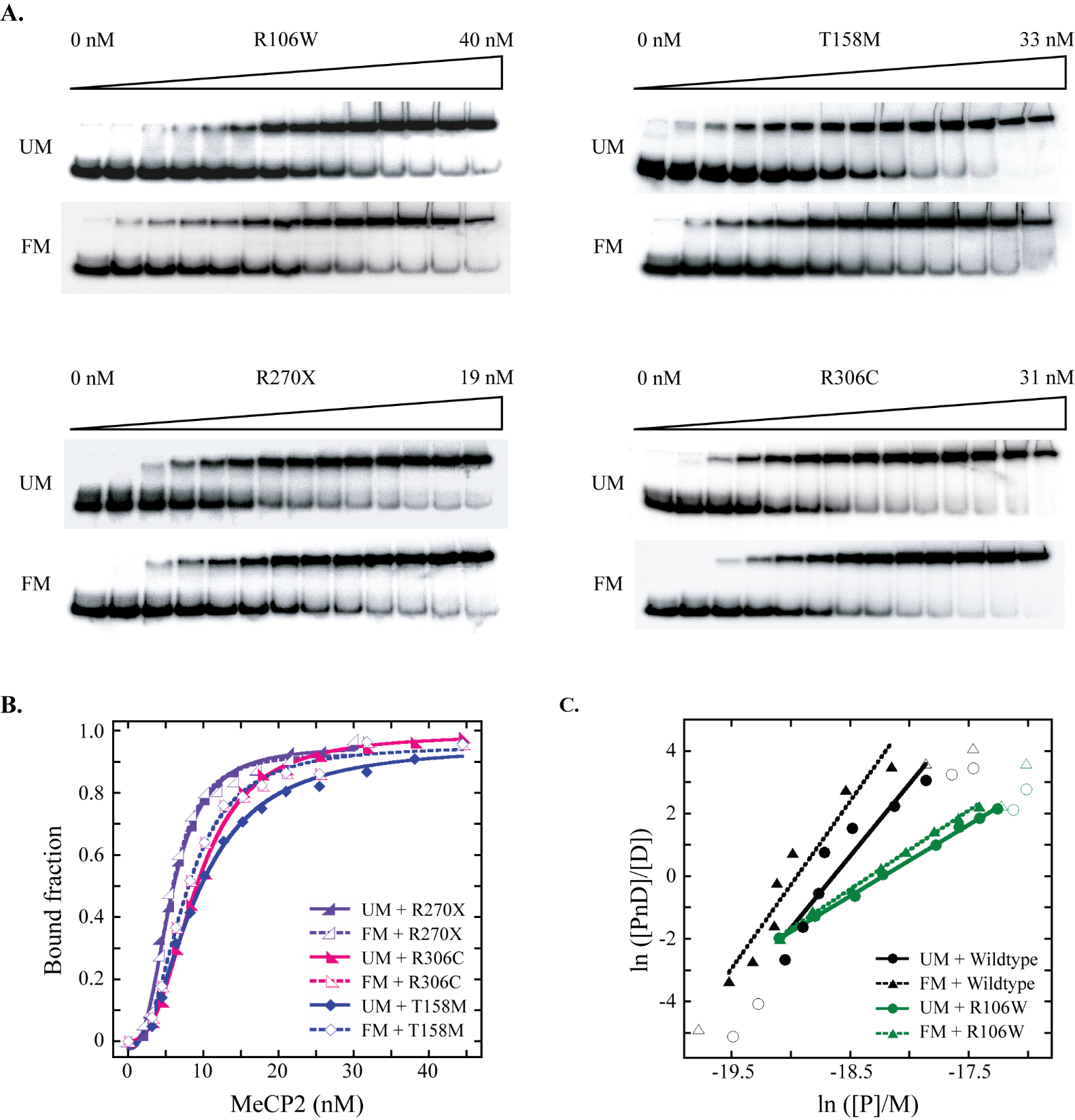


**Fig S11. MeCP2 binding to an unmethylated and fully methylated 195-bp DNA. A**, Representative electrophoretic mobility shift assays (EMSA) for MeCP2 variants binding to unmethylated (UM) or fully methylated (FM) 195 bp targets. A single sharp, high-molecular-weight complex is observed. **B.** Binding data from **A** were fit to a cooperative binding model to derive dissociation constants (*K_d_*) and Hill coefficients (h). Data represent the mean ± s.d. from at least three independent experiments. **C.** Data from the EMSA experiments were also used to create ln [P_n_D]/[D] vs ln[P] plots. Lines are least squares fits to each data set. Binding stoichiometry (n) and monomer-equivalent association constants (*K_mono_*) are given in **Table 6.** Data are the mean ± s.d. from at least three independent experiments.
